# Biomass-Derived Activated Biochars to CO_2_ Adsorption

**DOI:** 10.3390/molecules31172971

**Published:** 2026-08-25

**Authors:** Oscar de Almeida Neuwald, Ana Paula Prigol, Luiz Gustavo Tyska, Márcia Borghetti, Daniele Perondi, Marcelo Godinho

**Affiliations:** 1 Engineering of Processes and Technologies Post-Graduate Program, University of Caxias do Sul—UCS, Caxias do Sul 95070-560, Brazil; oaneuwald@ucs.br (O.d.A.N.); apprigol1@uca.br (A.P.P.); lgtyska@ucs.br (L.G.T.); mborghet@ucs.br (M.B.); dperondi1@ucs.br (D.P.); 2 UCSGRAPHENE Research and Development Unit, University of Caxias do Sul—UCS, Caxias do Sul 95070-560, Brazil

**Keywords:** biochar, carbon dioxide adsorption, activated carbon, biomass valorization

## Abstract

The development of low-cost and sustainable adsorbents for carbon dioxide (CO_2_) capture has gained increasing attention as a strategy to mitigate greenhouse gas emissions. In this study, activated biochars produced from babassu, elephant grass, and *Pinus elliottii* were evaluated as CO_2_ adsorbents after different activation treatments. The biochars were produced by slow pyrolysis at 400 °C and subsequently modified using three activation routes: steam activation, chemical activation with KOH, and KOH activation followed by acid washing. The materials were characterized by proximate analysis, specific surface area measurements, scanning electron microscopy coupled with energy-dispersive X-ray spectroscopy, and CO_2_ adsorption tests. Steam activation produced the highest specific surface areas, reaching 1270.53, 1027.28, and 907.87 m^2^ g^−1^ for babassu, elephant grass and *Pinus*, respectively. Despite the superior textural properties achieved through steam activation, the highest CO_2_ adsorption capacities were obtained for the samples subjected to chemical activation followed by acid washing. These results indicate that adsorption performance is governed not only by the development of surface area but also by pore accessibility and the surface chemistry of the adsorbent. Maximum adsorption capacities of 82.78, 81.19, and 85.24 mg g^−1^ were obtained for ACKAW B, ACKAW CE, and ACKAW P, respectively. Adsorption–desorption cycling experiments demonstrated regenerability and stable performance over repeated cycles. The results indicate that KOH activation followed by acid washing is an effective strategy for producing high-performance biochar-based adsorbents for CO_2_ capture.

## 1. Introduction

The continuous growth of the global population and the expansion of industrial activities have intensified the consumption of fossil fuels, resulting in a substantial increase in atmospheric carbon dioxide (CO_2_) emissions. As one of the primary greenhouse gases, CO_2_ plays a major role in global climate change, contributing to rising average temperatures, alterations in precipitation patterns, and increased frequency of extreme weather events. Atmospheric CO_2_ concentrations have increased substantially over recent decades and are expected to continue rising if mitigation strategies are not implemented [1,2]. Consequently, the development of efficient technologies for CO_2_ capture and mitigation has become a strategic priority in the transition toward a low-carbon economy.

Among the available carbon capture technologies, post-combustion capture has attracted considerable attention due to its potential integration into existing industrial systems. Within this context, absorption, membrane separation, and adsorption are the most extensively studied approaches. Although liquid absorption using amine-based solvents is currently the most mature technology, its large-scale application is limited by drawbacks such as solvent degradation, equipment corrosion, and high energy demand during regeneration [1,3]. Membrane processes offer operational simplicity but often suffer from limitations related to selectivity and sensitivity to contaminants [4]. In contrast, adsorption using porous solid materials combines relatively low regeneration energy requirements, high selectivity, operational flexibility, and the possibility of employing low-cost adsorbents derived from renewable resources [5,6].

Carbonaceous materials have emerged as promising adsorbents for CO_2_ capture because of their developed pore structures, tunable surface chemistry, thermal stability, and regeneration capability. Activated carbons, graphene derivatives, carbon molecular sieves, and biochars have all been investigated for this purpose. Among these materials, biochar has received increasing attention owing to its low production cost, renewable origin, and environmental benefits [7,8,9]. Biochar is a carbon-rich solid produced through the thermochemical conversion of biomass under oxygen-limited conditions, typically by pyrolysis [10,11]. In addition to offering a sustainable route for biomass valorization, biochar can simultaneously contribute to carbon sequestration and greenhouse gas mitigation [12,13].

The physicochemical properties of biochar strongly depend on both the feedstock characteristics and the pyrolysis conditions. Biomass composition, heating rate, residence time, and pyrolysis temperature directly influence the development of porosity, surface functional groups, and carbon content [14,15,16]. Biomasses such as babassu endocarp, elephant grass (*Pennisetum purpureum*), and *Pinus elliottii* wastes are particularly attractive feedstocks because of their high availability, renewable nature, and potential for conversion into high-value carbonaceous products [17,18,19]. Nevertheless, biochars produced at relatively low pyrolysis temperatures often exhibit limited surface area and underdeveloped microporosity, restricting their performance as CO_2_ adsorbents [20,21].

To overcome these limitations, different activation strategies have been employed to enhance the adsorption properties of biochars. Physical activation, commonly performed using steam or carbon dioxide at elevated temperatures, promotes the controlled gasification of the carbon matrix and the development of porous structures [22,23].

Chemical activation, particularly using potassium hydroxide (KOH), is recognized as one of the most effective methods for generating highly microporous carbon materials and increasing specific surface area [24,25,26]. However, KOH activation may leave significant amounts of residual inorganic species, including potassium-containing compounds such as K_2_CO_3_ and K_2_O, within the carbon matrix [27]. These residues can partially block micropores, reduce pore accessibility, and limit the effective use of adsorption sites. In addition, post-treatment procedures such as acid washing can remove residual inorganic species formed during activation, increasing pore accessibility and improving the effectiveness of adsorption sites [28]. As a result, the combination of KOH activation and subsequent demineralization has emerged as a promising route for the development of high-performance carbon adsorbents [29,30].

Several studies have demonstrated the potential of biomass-derived biochars for carbon capture applications [31,32,33]. Ref. [6] reported that wood- and straw-derived biochars exhibited CO_2_ adsorption capacities up to 0.97 mmol g^−1^, while [34] observed that increasing pyrolysis temperature enhanced both surface area development and CO_2_ uptake. Similarly, ref. [5] demonstrated that bamboo-derived biochar achieved adsorption capacities exceeding 2.5 mmol g^−1^ under optimized conditions. Despite these advances, direct comparisons among different biomass feedstocks processed under identical pyrolysis and activation conditions remain scarce.

Although numerous studies have investigated biomass-derived biochars for CO_2_ capture, most investigations focus on a single precursor or evaluate materials prepared under different activation conditions, making direct comparison difficult [35,36,37]. Biomasses differ considerably in lignocellulosic composition, mineral content, and carbonization behavior, which directly influence pore development and adsorption performance. Therefore, a systematic comparison of different biomass precursors subjected to identical pyrolysis, activation, and post-treatment conditions remains necessary to better understand the relationship between precursor characteristics and CO_2_ adsorption efficiency.

Therefore, this study aimed to investigate the influence of physical and chemical activation routes on the physicochemical properties and CO_2_ adsorption performance of biochars produced from babassu endocarp, elephant grass, and *Pinus elliottii*. The materials were characterized by proximate analysis, textural characterization, scanning electron microscopy coupled with energy-dispersive X-ray spectroscopy (SEM–EDS), and CO_2_ adsorption measurements. Emphasis was placed on correlating the structural and chemical modifications induced by the different activation routes with the CO_2_ adsorption performance, with the goal of identifying efficient and sustainable biochar-based adsorbents for carbon capture applications.

## 2. Results and Discussion

### 2.1. Biomass Pyrolysis

**Figure 1:**
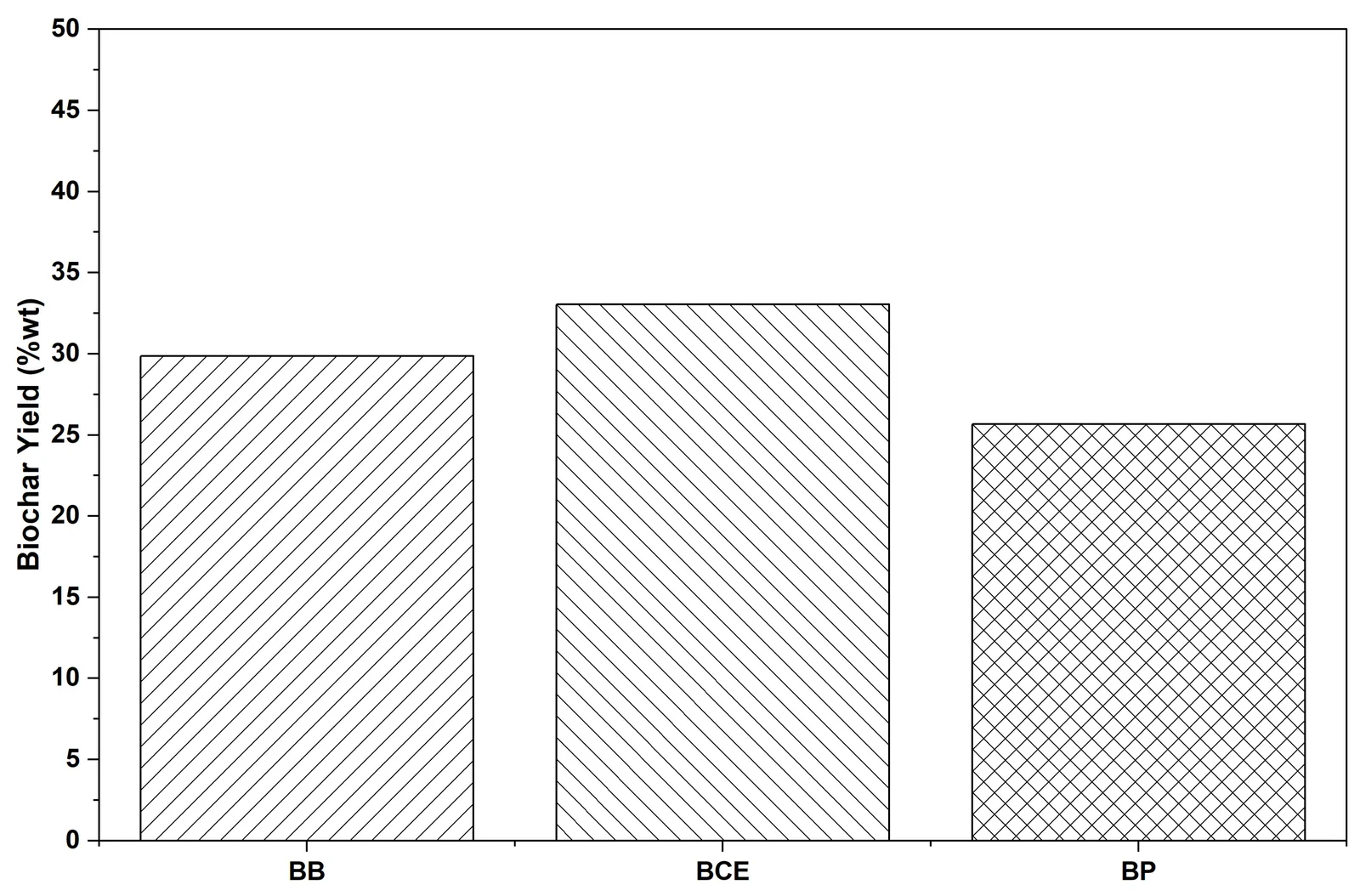
Biochar yield from the pyrolysis of babassu (BB), elephant grass (BCE), and *Pinus* (BP).

The biochar yields obtained from the pyrolysis of babassu, elephant grass, and *Pinus* at 400 °C are presented in Figure 1. The biochar yields were 29.85 wt.% for babassu (BB), 33.04 wt.% for elephant grass (BCE), and 25.68 wt.% for *Pinus* (BP). These values are consistent with the typical range reported for slow pyrolysis processes, which generally yield between 25 and 35 wt.% of biochar [38,39].

The differences observed among the biomasses can be attributed to variations in their lignocellulosic composition, particularly lignin, cellulose, hemicellulose, and extractives contents, which directly influence the thermal decomposition behavior during pyrolysis [40]. Biomass feedstocks with higher thermal stability tend to retain a larger fraction of carbon in the solid phase, contributing to greater biochar yields.

The relatively high yields obtained are also associated with the moderate pyrolysis temperature employed in this study. At 400 °C, thermal decomposition remains limited compared to higher temperatures, favoring carbon retention in the solid fraction while minimizing the formation of condensable vapors and permanent gases.

The proximate analysis results are presented in Table 1. Moisture contents ranged from 5.40 to 8.09 wt.%, with the highest value observed for BCE. Since moisture can negatively affect CO_2_ adsorption by competing for adsorption sites and partially blocking pore structures, all materials were dried before adsorption experiments.

Volatile matter contents remained relatively similar among the biochars, ranging from 25.23 wt.% for BB to 27.18 wt.% for BP, indicating the presence of residual organic compounds that were not completely removed during pyrolysis.

More pronounced differences were observed for ash and fixed carbon contents. BCE exhibited the highest ash content (18.43 wt.%), whereas BB and BP presented significantly lower values of 4.71 wt.% and 1.87 wt.%, respectively. The elevated ash content of BCE is consistent with the naturally high mineral concentration found in elephant grass biomass [17,41]. Although certain mineral constituents may contribute to CO_2_ uptake through chemisorption mechanisms [42], excessive ash levels are generally undesirable because they can reduce the available surface area and obstruct the porous structure of the adsorbent.

Regarding fixed carbon, BB and BP exhibited values close to 70 wt.% (70.06 and 70.99 wt.%, respectively), while BCE showed a lower value of 55.02 wt.%. High fixed-carbon contents are generally associated with greater degrees of carbonization, improved thermal stability, and enhanced potential for pore development during activation processes. These characteristics are particularly desirable for applications involving CO_2_ capture.

Proximate analysis suggests that babassu and *Pinus* biochars possess more favorable characteristics for subsequent activation and adsorption applications due to their combination of high fixed-carbon content and low ash concentration.

### 2.2. Chemical Activation

#### 2.2.1. CO_2_ Adsorption Performance of Activated Biochars

**Figure 2:**
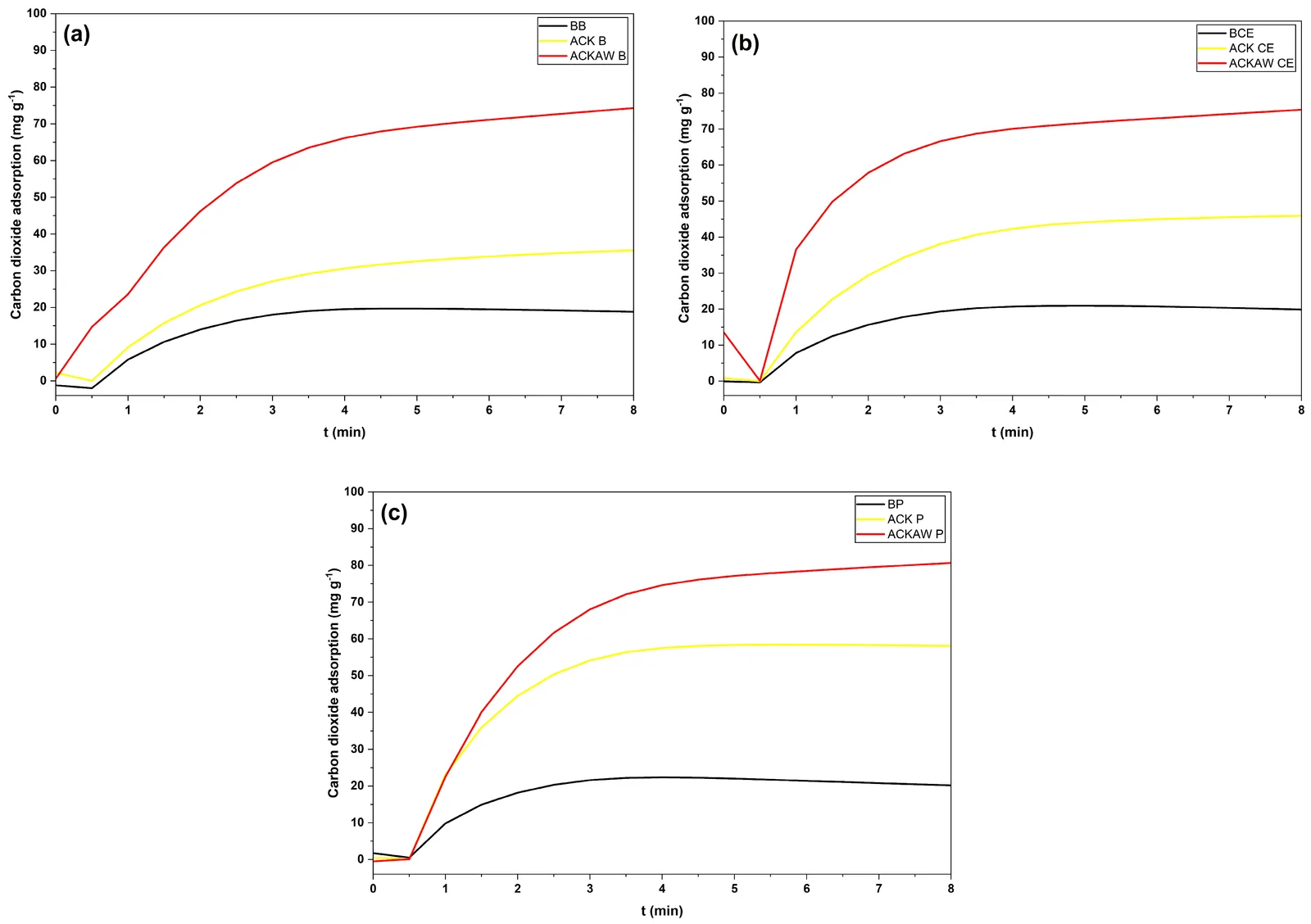
Carbon dioxide adsorption on KOH-activated samples with and without acid washing: babassu (**a**), elephant grass (**b**), and *Pinus* (**c**).

The CO_2_ adsorption of biochars produced from babassu, elephant grass, and *Pinus*, as well as their chemically activated carbon, are presented in Figure 2. The adsorption profiles reveal a similar adsorption pattern for all materials, characterized by a rapid uptake during the first minutes of contact, followed by a gradual decrease in the adsorption rate until equilibrium was approached.

For all biomasses evaluated, chemical activation significantly enhanced the CO_2_ adsorption capacity compared to their respective raw biochars. Moreover, the additional acid washing step after KOH activation promoted a further increase in adsorption performance, indicating that the removal of residual inorganic compounds contributed to the development and accessibility of the porous structure.

In the case of babassu-derived materials (Figure 2a), the biochar (BB) exhibited the lowest adsorption capacity. A rapid increase in adsorption was observed between 0.5 and 3 min, followed by stabilization near the equilibrium condition. The KOH-activated sample (ACK B) exhibited substantially higher adsorption, corresponding to an increase of nearly 90% relative to BB. The acid-washed activated biochar (ACKAW B) demonstrated the highest performance among the babassu-based materials, which represents nearly a fourfold increase compared to the untreated biochar.

A similar trend was observed for elephant grass-derived biochars (Figure 2b). The biochar (BCE) reached equilibrium at 21.08 mg g^−1^, whereas the activated sample (ACK CE) achieved 46.46 mg g^−1^. Following acid washing, the adsorption capacity increased further to 81.19 mg g^−1^. Notably, the ACKAW CE sample exhibited a very steep adsorption increase during the first minute, indicating a larger number of readily accessible adsorption sites and enhanced mass transfer of CO_2_ within the porous network.

The most pronounced enhancement was observed for *Pinus*-derived biochars (Figure 2c). The untreated biochar (BP) presented a maximum adsorption 22.51 mg g^−1^, while activation with KOH increased adsorption to 58.68 mg g^−1^. The acid-washed material (ACKAW P) achieved the highest adsorption capacity among all samples, reaching 85.24 mg g^−1^ at the end of the adsorption experiment. These values indicate that *Pinus* biomass responded more effectively to the activation procedures, resulting in a highly developed pore structure suitable for CO_2_ capture.

The additional increase in adsorption following HCl washing indicates that residual inorganic species remained within the activated biochars after carbonization and activation. These species may partially block pore entrances or occupy active adsorption sites. Acid treatment effectively removes residual potassium compounds, ashes, and mineral matter, increasing pore accessibility and exposing additional adsorption sites.

#### 2.2.2. Micropore Volume

**Figure 3:**
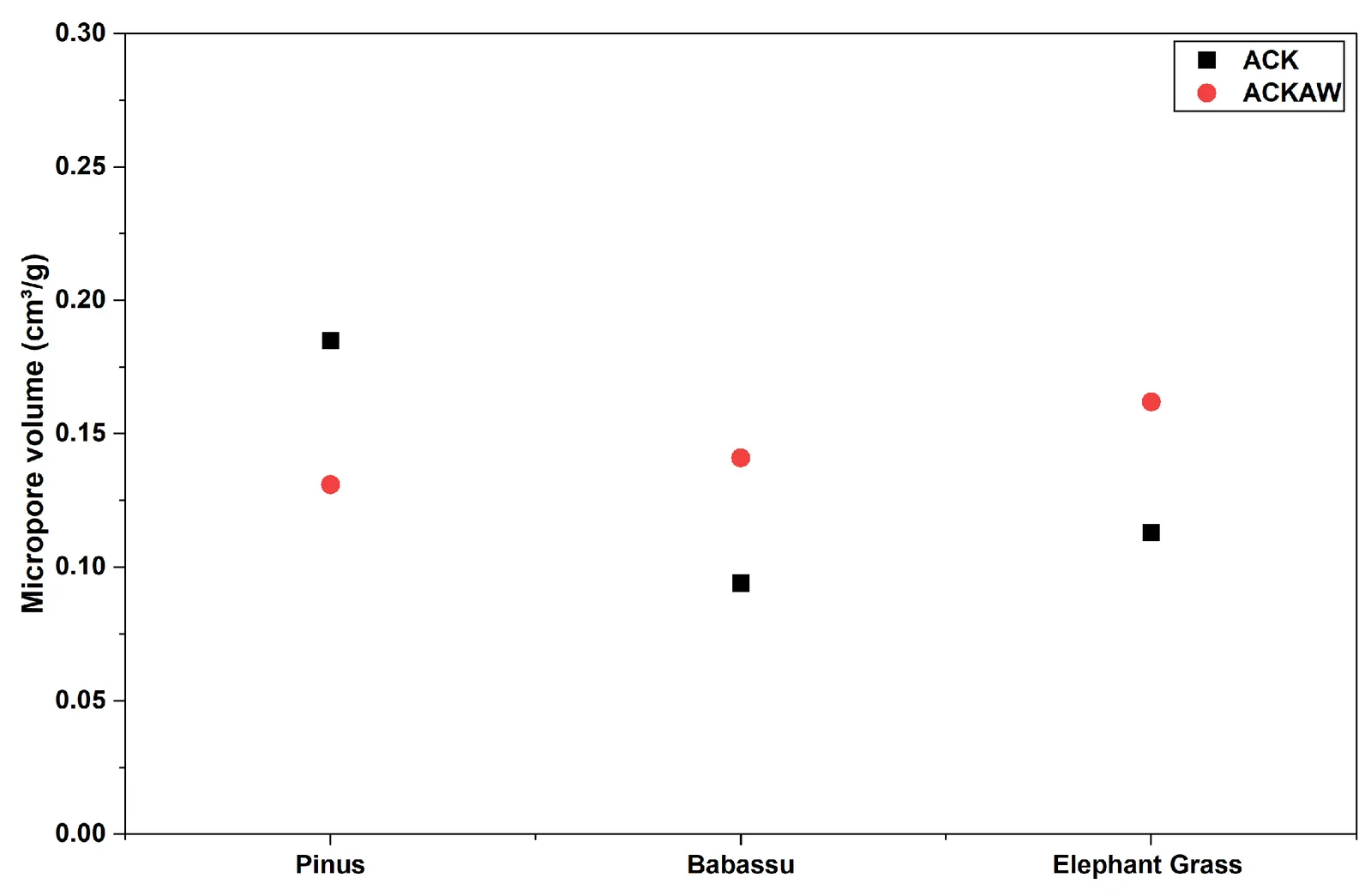
Micropore volume of KOH-activated biochars from *Pinus*, babassu, and elephant grass, with and without HCl acid washing.

As shown in Figure 3, the non-washed ACK P exhibited the highest micropore volume (0.18 cm^3^ g^−1^), whereas ACK B and ACK CE presented lower values (0.09 and 0.11 cm^3^ g^−1^, respectively). After HCl washing, a different trend emerged: micropore volume decreased in ACKAW P (0.13 cm^3^ g^−1^) but increased in ACKAW B (0.14 cm^3^ g^−1^) and ACKAW CE (0.16 cm^3^ g^−1^).

These results suggest that the influence of acid washing depends strongly on the mineral composition and carbon structure of each precursor. For ACKAW B and ACKAW CE, the increase in micropore volume indicates that residual potassium compounds and ash particles were occupying or blocking internal pores generated during KOH activation. The dissolution of these inorganic species by HCl exposed previously inaccessible micropores, thereby increasing the measurable micropore volume.

Conversely, the decrease observed for ACKAW P suggests that part of the pore system classified as microporous before washing may have been enlarged after mineral removal, may have reflected improved accessibility and exposure of previously blocked pores. This interpretation is supported by the average pore width results discussed below.

KOH activation is known to produce metallic potassium and potassium carbonate intermediates that penetrate the carbon matrix and create porosity through carbon gasification reactions. Subsequent acid leaching removes these species and may significantly alter the final pore structure [43,44].

#### 2.2.3. Average Pore Width

**Figure 4:**
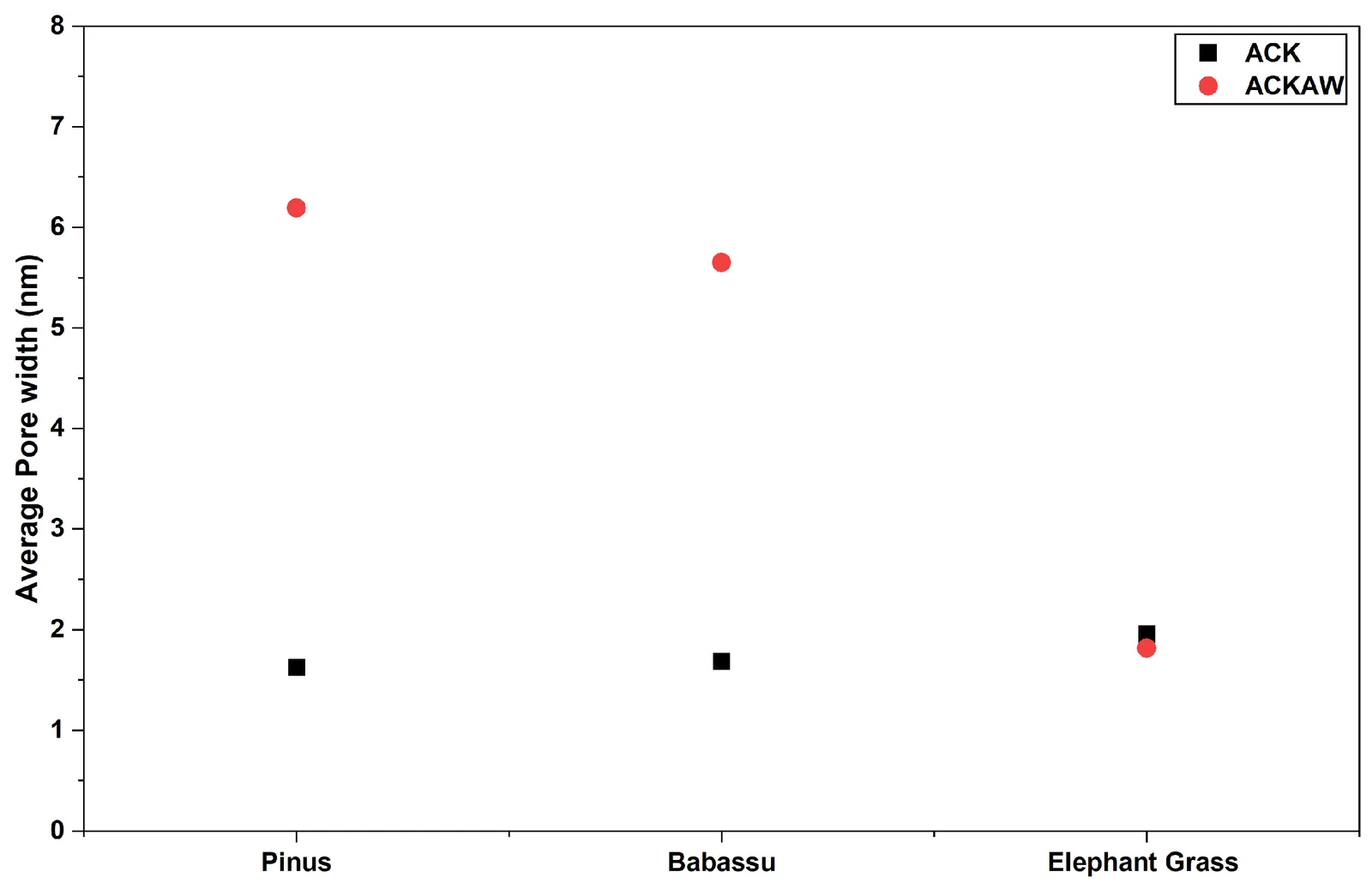
Average Pore Width of KOH-activated carbons from *Pinus*, babassu, and elephant grass, with and without HCl acid washing.

Figure 4 shows that acid washing substantially increased average pore width for ACK P and ACK B. Values increased from approximately 1.63–1.69 nm to 5.65–6.19 nm. In contrast, ACK CE exhibited 1.96 nm, remaining relatively constant (1.82 nm) after washing.

This behavior indicates that the pore system of ACK P and ACK B underwent increased accessibility of previously blocked pores after the removal of mineral deposits. The ash and potassium-containing species generated during activation likely occupied part of the pore volume and restricted pore diameters. Their removal exposed wider channels and increased pore accessibility.

ACK CE maintained nearly the same pore width even after acid treatment, suggesting that its pore structure generated during KOH activation was more stable and less dependent on mineral blockage. Instead of promoting pore enlargement, acid washing mainly increased pore accessibility while preserving narrow porosity.

From a CO_2_ capture perspective, this distinction is particularly important. Although larger pores facilitate gas diffusion, narrow micropores provide stronger adsorption potential due to overlapping interactions from opposing pore walls. Consequently, maintaining relatively narrow pores is often advantageous for CO_2_ adsorption, especially at low pressures [45].

#### 2.2.4. Micropore Surface Area

**Figure 5:**
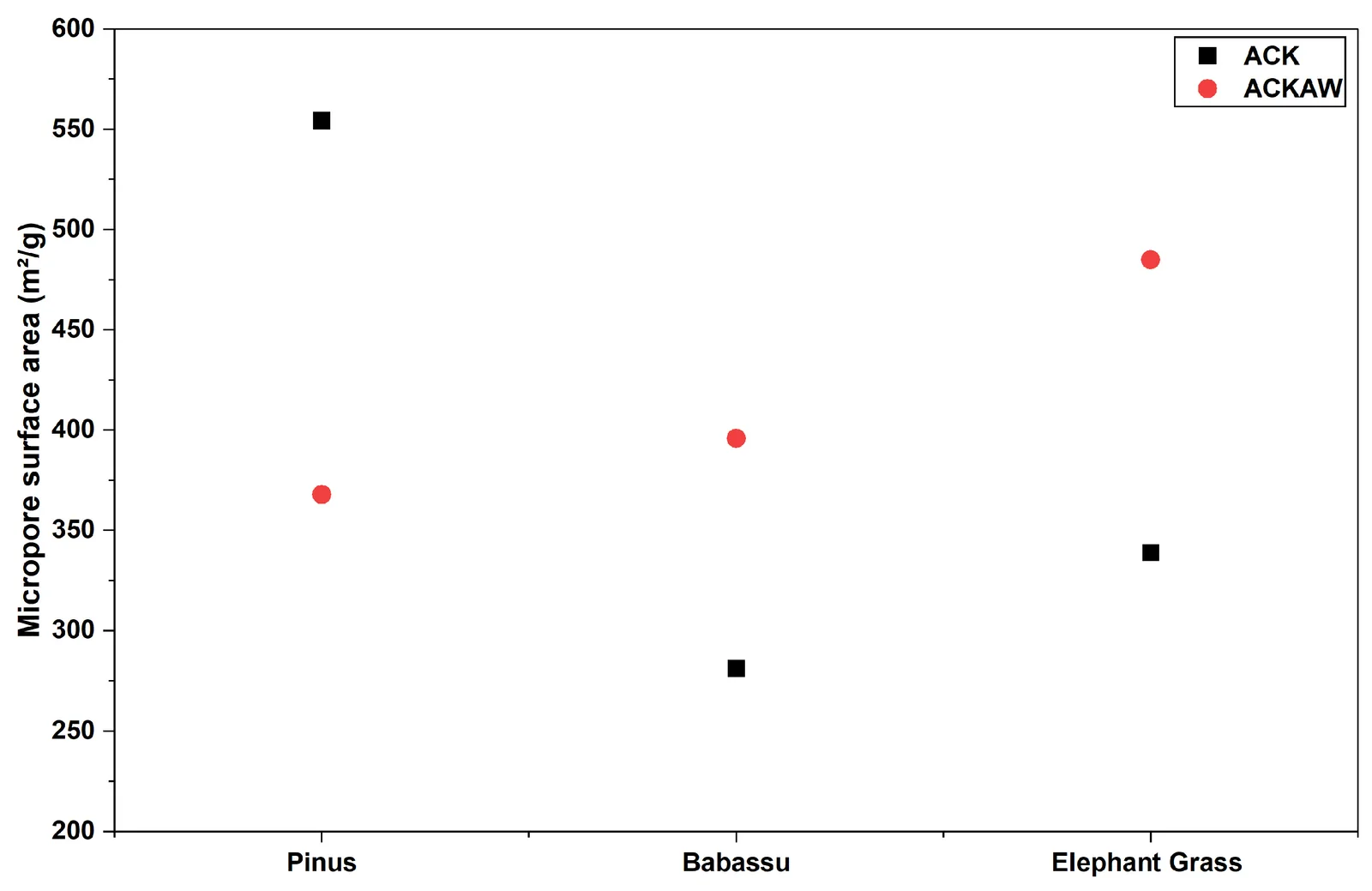
Micropore surface area of KOH-activated biochars from *Pinus*, babassu, and elephant grass, with and without HCl acid washing.

The results for micropore surface area (Figure 5) provide further evidence of the structural changes induced by acid washing. Before washing, ACK P possessed the highest micropore area (554.41 m^2^ g^−1^), followed by ACK CE (338.99 m^2^ g^−1^) and ACK B (281.29 m^2^ g^−1^). After HCl treatment, ACK P experienced a pronounced reduction to approximately 367.86 m^2^ g^−1^, whereas ACK B and ACK CE exhibited substantial increases, reaching about 395.87 and 484.93 m^2^ g^−1^, respectively.

The reduction in ACK P is consistent with the pore widening observed previously. When previously inaccessible pore domains become accessible, total micropore surface area may decrease because surface area is inversely related to pore diameter. Thus, despite losing part of its microporous surface, *Pinus* exhibited greater pore accessibility.

The opposite behavior observed in ACK B and ACK CE indicates that acid washing exposed additional microporous surfaces previously blocked by mineral residues. The fact that Elephant Grass simultaneously displayed increased micropore volume, increased micropore area, and nearly unchanged pore width strongly suggests effective cleaning of existing pores without significant structural collapse or widening.

Among the evaluated materials, Elephant Grass exhibited the most favorable combination of micropore volume and micropore surface area after washing, indicating highly efficient development of accessible adsorption sites.

#### 2.2.5. Influence of Activation and Acid Washing on Ash Content

**Figure 6:**
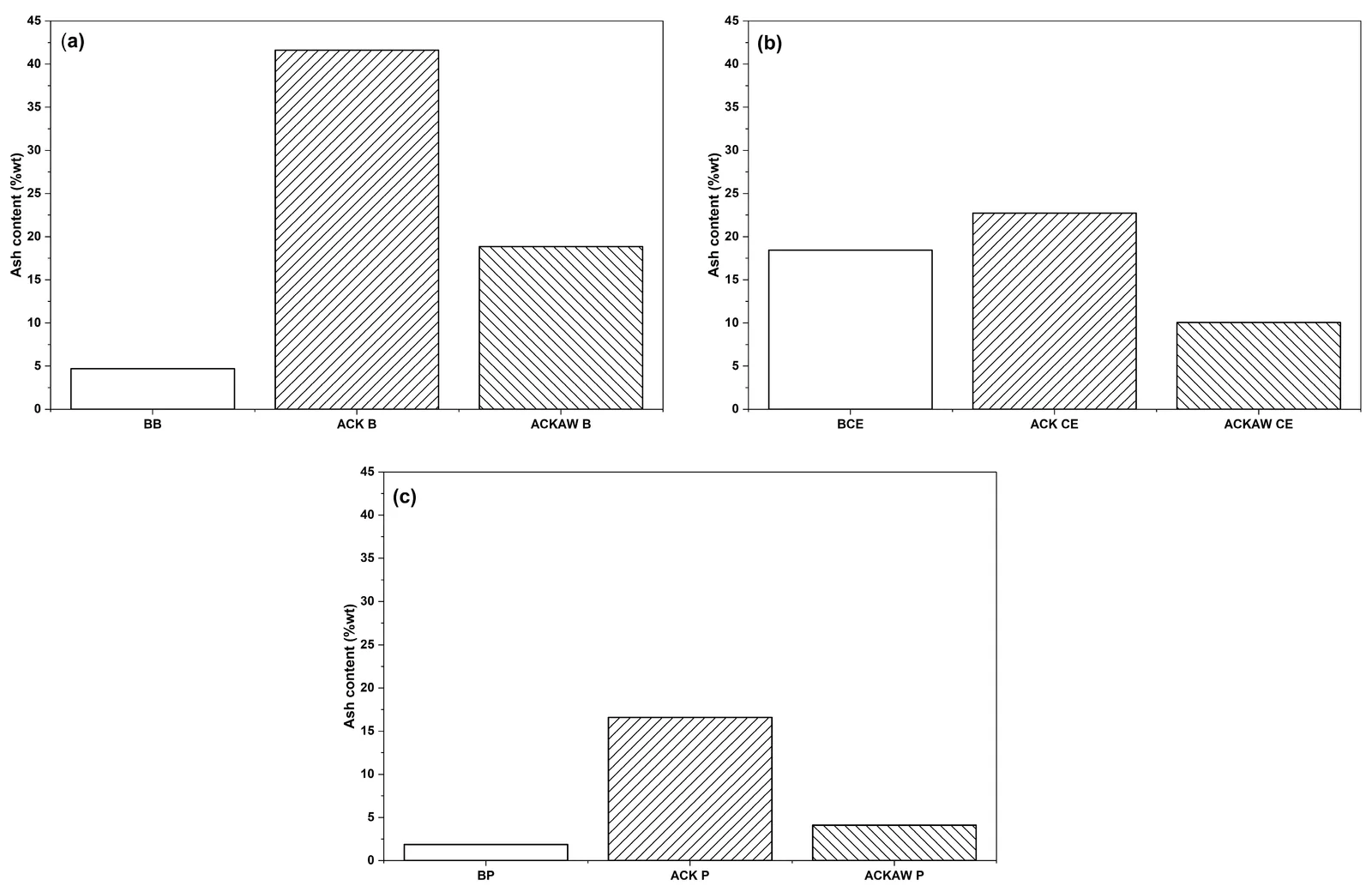
Ash content of biochars and activated biochar from babassu (**a**), elephant grass (**b**), and *Pinus* (**c**).

The ash contents of the original biochars and activated biochars are shown in Figure 6. Significant variations were observed after KOH activation and acid washing, demonstrating that these treatments strongly affect the inorganic fraction of the biochars.

The ash contents reached 41.63 wt.% for ACK B, 22.74 wt.% for ACK CE, and 16.60 wt.% for ACK P. During chemical activation, the reaction between KOH and carbon generates potassium-containing inorganic species such as K_2_CO_3_ and K_2_O [25,46]. Because these compounds remain within the porous network after thermal treatment, the mineral fraction of the material increases substantially.

Subsequent washing with HCl effectively reduced the ash content of all chemically activated biochars. The values decreased to 18.85 wt.% for ACKAW B, 10.06 wt.% for ACKAW CE, and 4.11 wt.% for ACKAW P. These results demonstrate the effectiveness of acid washing in removing residual potassium compounds and other inorganic constituents generated during activation.

For *Pinus* and babassu, the final ash contents remained slightly higher than those of the original biochars, suggesting that a portion of the mineral matter remained trapped within the carbon matrix even after acid treatment. In contrast, the ash content of ACKAW CE became lower than that of the original BCE sample, indicating a more efficient removal of inorganic species from elephant-grass-derived material.

The substantial reduction in ash content after acid washing is particularly relevant for adsorption applications, since the removal of mineral deposits can increase pore accessibility and improve the availability of adsorption sites for CO_2_ molecules.

#### 2.2.6. EDS Analysis

The elemental composition of the biochars and activated biochars was investigated by energy-dispersive X-ray spectroscopy (EDS), and the results are presented in Figures S1–S3 (Supplementary Materials) and Table 2. Carbon was the predominant element in all samples, confirming the successful conversion of the raw biomass into carbonaceous materials during pyrolysis and activation processes.

The chemical activation with KOH significantly altered the elemental composition of the materials. The most notable change was the increase in potassium concentration, indicating the retention of potassium-containing species (K_2_CO_3_ or K_2_O) formed during the activation process. These species are commonly associated with the reactions between KOH and the carbon matrix, which are responsible for pore development and structural modification. The higher mineral content detected by EDS is consistent with the increase in ash content observed after chemical activation.

Subsequent acid washing with HCl effectively reduced the concentration of potassium and other inorganic elements. This behavior confirms the removal of residual activating-agent-derived species and mineral compounds that remained attached to the carbon surface after activation. Consequently, the relative carbon content increased in all washed samples, indicating a purification of the carbon matrix and a reduction in the inorganic fraction.

Samples subjected to KOH activation exhibited a more pronounced distribution of potassium-containing regions, whereas acid-washed samples displayed a more homogeneous carbon-rich surface with a substantial reduction in mineral-associated signals. These results agree with the ash content measurements and suggest that the acid treatment improved the accessibility of the porous structure by removing inorganic deposits that could partially block pore entrances.

The EDS and ash analyses are consistent with the morphological observations obtained by SEM. Samples subjected to KOH activation exhibited high potassium contents (7.5–9.6 wt.%) and elevated ash contents (16.60–41.63 wt.%), indicating the retention of inorganic activation residues within the carbon matrix. After HCl washing, potassium concentrations decreased to negligible levels, accompanied by substantial reductions in ash content. The cleaner carbon surfaces observed by SEM after acid treatment suggest the removal of mineral deposits that previously obstructed pore entrances. This combined evidence supports the conclusion that demineralization improved pore accessibility and contributed directly to the higher CO_2_ adsorption capacities observed for the ACKAW samples.

#### 2.2.7. Adsorption Energy

**Figure 7:**
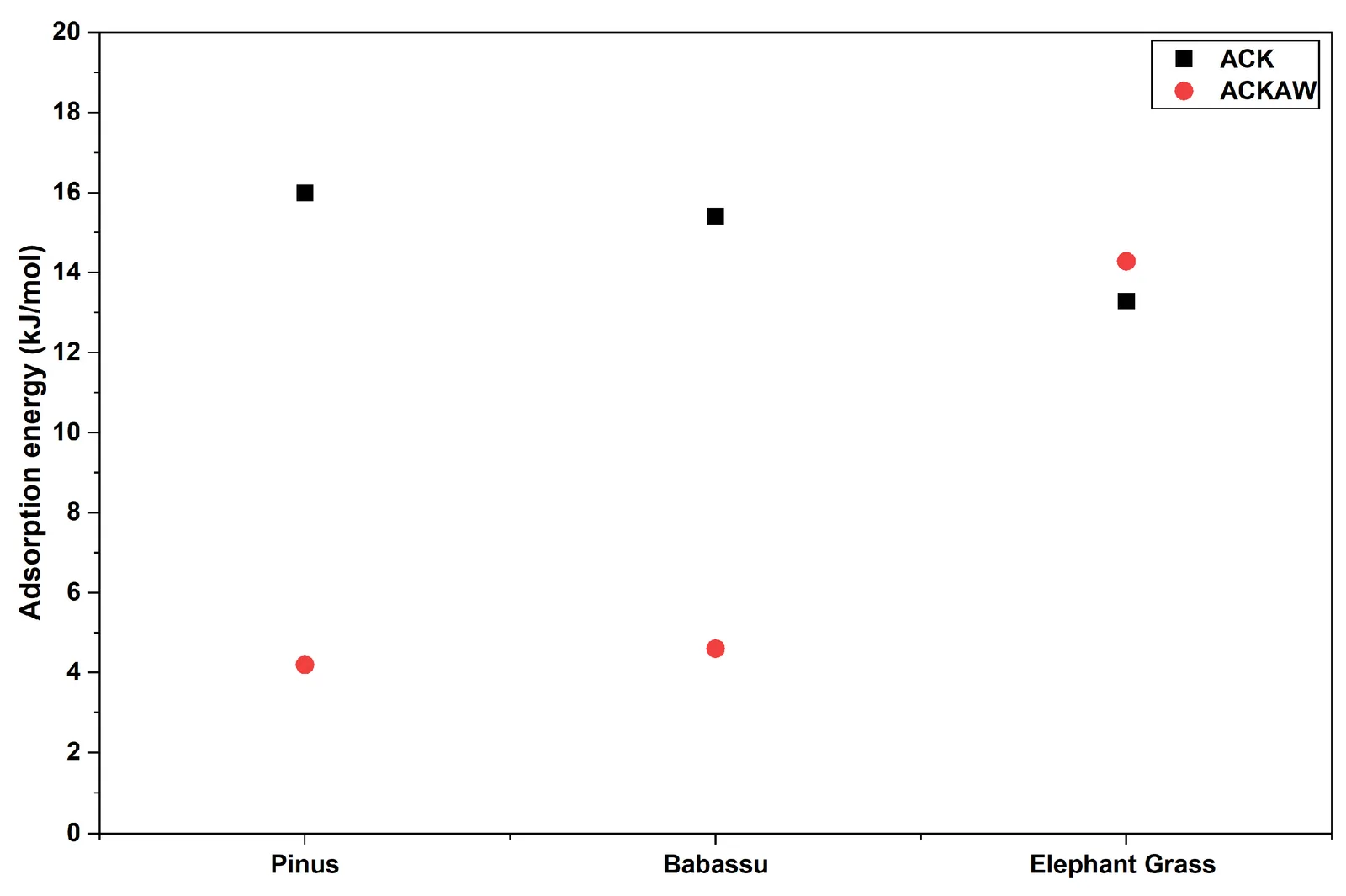
Adsorption energy of KOH-activated biochars from *Pinus*, babassu, and elephant grass, with and without HCl acid washing.

Figure 7 reveals an interesting effect of acid washing on adsorption energy. The non-washed samples displayed adsorption energies ranging from approximately 13 to 16 kJ mol^−1^, with *Pinus* presenting the highest value (15.992 kJ mol^−1^). After acid washing, ACKAW P and ACKAW B experienced a decrease to 4.199–4.602 kJ mol^−1^, whereas ACKAW CE maintained a value of 14.285 kJ mol^−1^.

This result suggests that adsorption sites present in *Pinus* and Babassu before washing were associated, at least partially, with mineral components or potassium-containing species remaining after activation. Once these species were removed, the average adsorption energy declined substantially.

In contrast, Elephant Grass retained high adsorption energy even after demineralization, indicating that its adsorption performance is predominantly governed by intrinsic pore structure rather than by inorganic residues. The preservation of narrow pores following washing likely contributes to stronger confinement effects and consequently stronger adsorbate–surface interactions.

#### 2.2.8. Maximum CO_2_ Adsorption Capacity

**Figure 8:**
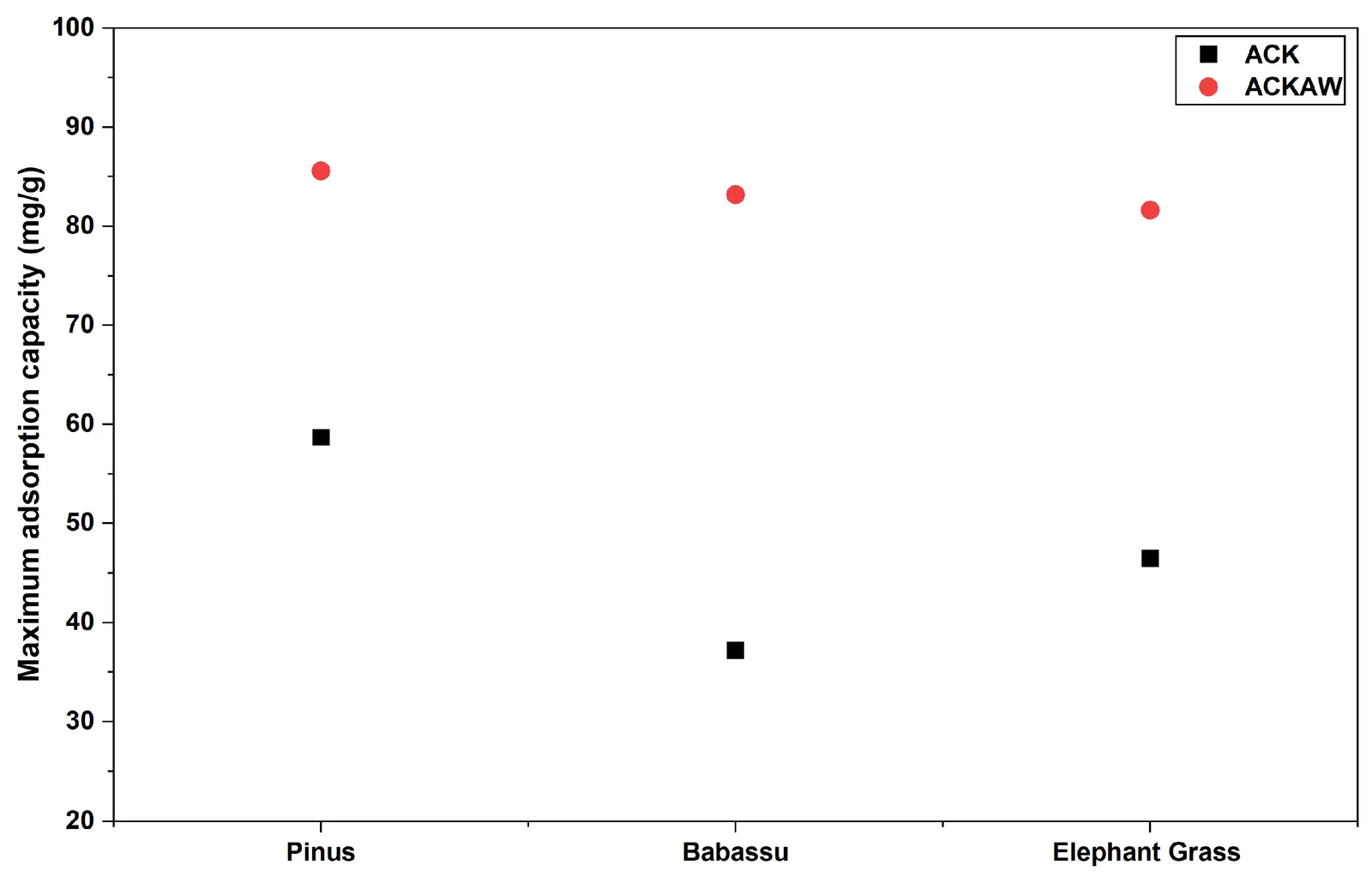
Maximum CO_2_ adsorption capacity of KOH-activated carbons from *Pinus*, Babassu, and Elephant grass, with and without HCl acid washing.

The result of maximum CO_2_ adsorption capacity is presented in Figure 8. All acid-washed samples exhibited substantially higher maximum adsorption capacities than their corresponding non-washed counterparts. Capacities increased from 58.68 to 85.24 mg g^−1^ for *Pinus*; 37.17 to 82.78 mg g^−1^ for Babassu; 46.46 to 81.19 mg g^−1^ for Elephant Grass.

The improvement was particularly significant for Babassu and Elephant Grass, whose adsorption capacities nearly doubled following HCl treatment. The increase in CO_2_ uptake occurred even when micropore area decreased, as observed for *Pinus*. This finding demonstrates that adsorption performance cannot be explained solely by surface area. Instead, accessibility of adsorption sites and reduction of inorganic impurities appear to play a decisive role.

The acid washing step reduced ash content and removed potassium compounds that otherwise occupied pore volume and contributed to inert mass. Consequently, a greater fraction of the carbon structure became available for interaction with CO_2_ molecules. Similar effects have been reported for KOH-activated biochars where acid washing enhanced adsorption performance despite modest changes in textural parameters [47].

The combined results reveal that the adsorption capacity is primarily controlled by the balance between accessible microporosity and pore size distribution rather than by a single textural parameter.

For *Pinus*, acid washing reduced micropore volume and micropore area but increased pore accessibility, resulting in the highest adsorption capacity among all samples. For Babassu, the simultaneous increase in micropore volume, pore width, and micropore area produced an enhancement in adsorption capacity. Elephant Grass exhibited the most balanced behavior. Acid washing increased micropore volume and micropore area while preserving narrow pore widths and high adsorption energy. This combination generated one of the highest adsorption capacities and suggests that Elephant Grass developed the most efficient pore architecture for CO_2_ capture.

Overall, the results demonstrate that KOH activation successfully generated a porous carbon framework, while HCl washing acted as a critical purification step that removed ash and residual potassium species, increased accessibility of the porous network, and significantly enhanced CO_2_ adsorption. The superior performance of the washed materials confirms that demineralization is essential for maximizing the benefits of KOH activation and obtaining high-performance biochars for carbon capture applications [48].

The adsorption capacities obtained for the acid-washed KOH-activated biochars (82.78 mg g^−1^ for ACKAW B, 81.19 mg g^−1^ for ACKAW CE, and 85.24 mg g^−1^ for ACKAW P) compare favorably with values reported for several biomass-derived adsorbents in the literature. Biochars produced from peanut shell pyrolysis under optimized conditions achieved a maximum CO_2_ adsorption capacity of 64 mg g^−1^, while non-activated biochars derived from butiá pomace reached 66.43 mg g^−1^ at 25 °C and 1 bar [33,49]. Similarly, wood-pellet biochars activated by steam and KOH presented maximum adsorption capacities of 38.84 and 50.73 mg g^−1^, respectively [50]. Activated biochars produced from heavy-metal-contaminated biomass exhibited a maximum uptake of 2.10 mmol g^−1^ (approximately 92.4 mg g^−1^ of CO_2_), which is in the same range as the best materials developed in the present study [31]. A commercial carbon molecular sieve specifically engineered for PSA applications showed a higher adsorption capacity of 2.90 mmol g^−1^ (approximately 127.6 mg g^−1^), which can be attributed to its highly optimized microporous structure and industrial-grade manufacturing process [51]. Nevertheless, the adsorption capacities achieved by the acid-washed biochars are remarkably close to those of more sophisticated commercial adsorbents, while being produced from abundant renewable biomass residues. Therefore, the results demonstrate that the combination of KOH activation and acid washing is an effective strategy for producing low-cost and sustainable adsorbents with competitive CO_2_ capture performance.

### 2.3. Physical Activation

#### 2.3.1. Steam Activation on CO_2_ Adsorption Performance

**Figure 9:**
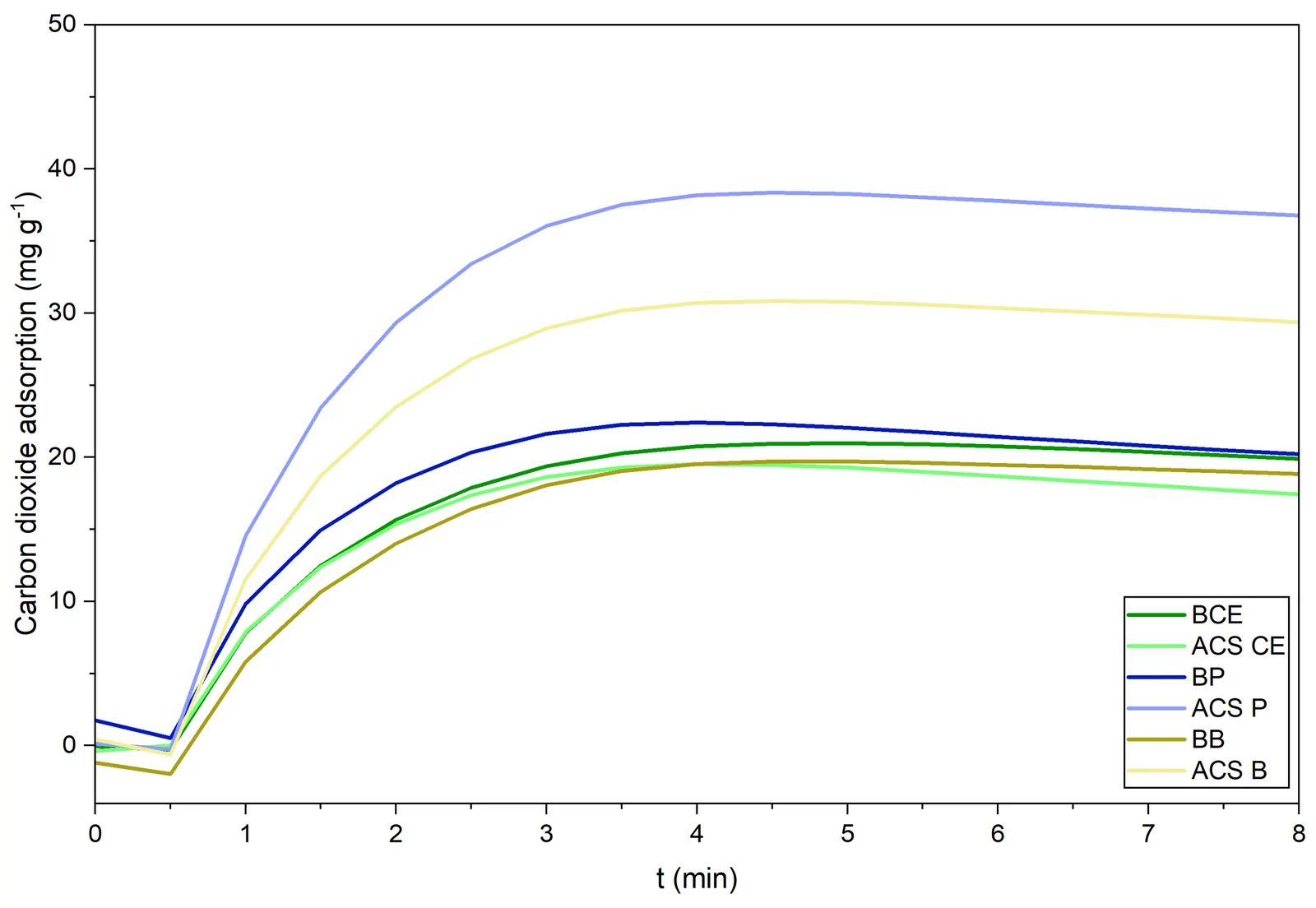
Carbon dioxide adsorption on steam-activated samples.

The CO_2_ adsorption curves (Figure 9) revealed that both the biomass precursor and the steam activation process significantly affected the adsorption behavior of the carbonaceous materials. All samples exhibited a similar profile, characterized by an initial rapid adsorption stage during the first minutes of contact followed by a plateau region after approximately 4 min, indicating the attainment of adsorption equilibrium.

Among the biochars, the material derived from *Pinus* displayed the highest CO_2_ adsorption capacity, reaching approximately 22 mg g^−1^, followed by the BCE and the BB, which achieved maximum adsorption capacities close to 21 and 20 mg g^−1^, respectively. These differences suggest that the intrinsic characteristics of the precursor biomass influenced pore formation during pyrolysis. The superior performance of BP indicates the development of a more favorable porous architecture for CO_2_ adsorption, likely associated with a higher fraction of micropores and a more accessible internal surface.

Steam activation substantially enhanced the adsorption performance of the babassu- and *Pinus*-derived biochars. For the *Pinus* precursor, the maximum adsorption capacity increased from 22.51 mg g^−1^ for BP to 38.54 mg g^−1^ for ACS P, corresponding to an increase of nearly 70%. Similarly, the adsorption capacity of the babassu-derived biochar increased from 19.80 mg g^−1^ for BB to 31 mg g^−1^ for ACS B, representing an improvement close to 60%. These results indicate that steam activation effectively promoted pore development and generated new adsorption sites in both materials.

The performance of ACS P suggests that the *Pinus*-derived carbon possesses a carbon matrix particularly suitable for micropore development during activation. The higher adsorption capacity observed for this material likely reflects an increased volume of micropores, which are recognized as the most effective pore domains for CO_2_ capture. Therefore, the substantial improvement observed after steam activation suggests that the activation process generated an optimized porous structure for CO_2_ adsorption in the *Pinus*-derived carbon.

In contrast, activation had a negligible effect on the elephant-grass-derived biochar. The adsorption capacity of ACS CE (19.62 mg g^−1^) remained lower than that of BCE, indicating that steam activation did not improve the CO_2_ capture performance of this precursor. This behavior may be attributed to excessive widening of micropores during activation, resulting in the formation of larger pores that are less effective for CO_2_ adsorption. Therefore, a reduction in the fraction of narrow micropores may explain the absence of performance enhancement after activation. Similar observations have been reported for biochar-derived activated carbons, where increases in surface area were not necessarily accompanied by increased CO_2_ adsorption capacity due to unfavorable modifications in pore size distribution [52,53].

While activation significantly improved the adsorption performance of *Pinus* and babassu, the same treatment was ineffective for elephant grass. These findings indicate that the response of biomass-derived carbons to activation cannot be generalized and must be individually optimized according to the characteristics of each precursor.

#### 2.3.2. Average Pore Width and Micropore Volume

**Figure 10:**
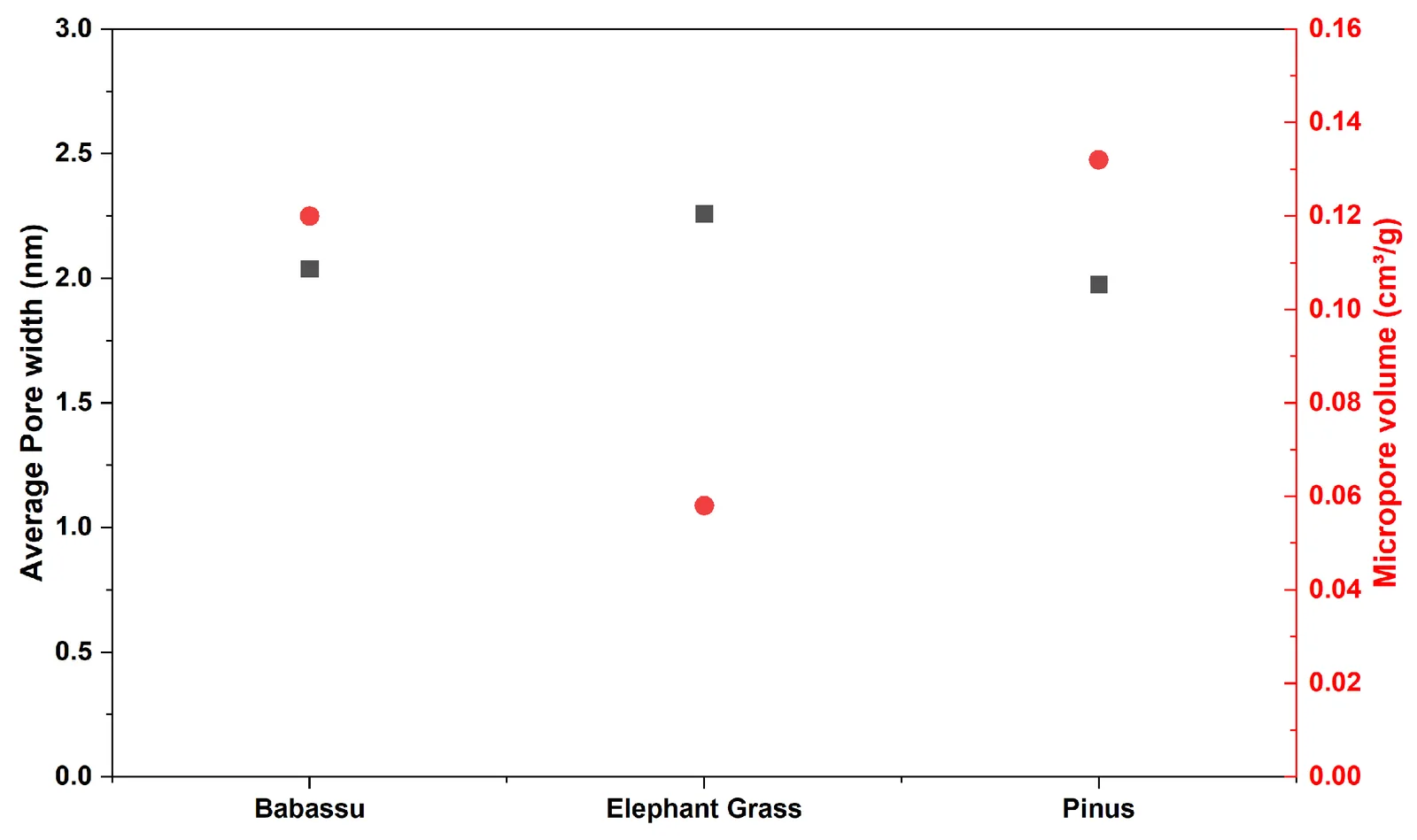
Average pore width versus micropore volume of steam-activated samples.

The average pore width and micropore volume are shown in Figure 10. Significant differences were found among the biomasses investigated. Elephant Grass exhibited the largest average pore width (2.26 nm), followed by Babassu (2.04 nm) and *Pinus* (1.97 nm). However, the micropore volume showed an opposite tendency. *Pinus* presented the highest micropore volume (0.13 cm^3^ g^−1^), followed by Babassu (0.12 cm^3^ g^−1^), whereas Elephant Grass exhibited a lower value (0.06 cm^3^ g^−1^).

This inverse relationship suggests that steam activation affected the carbon matrices differently. In Elephant Grass, the gasification process appears to have promoted a stronger widening of existing pores, converting part of the microporous structure into larger pores. Therefore, although the material developed wider pore channels, the total volume associated with micropores decreased.

In contrast, *Pinus* maintained a narrower pore structure while preserving a larger microporous volume. This indicates that steam activation generated porosity without excessive pore enlargement, favoring the retention of microporous domains that are generally regarded as the most important structures for CO_2_ adsorption.

The behavior of Babassu was intermediate, combining relatively high micropore volume with moderate average pore width, suggesting a balanced development between pore formation and pore widening.

From a mechanistic perspective, steam activation proceeds through progressive carbon gasification at active sites on the carbon surface. Initially, micropores are formed and expanded; however, excessive gasification can merge neighboring pores and generate wider channels. Therefore, maintaining a high micropore volume while minimizing excessive widening is often critical for effective CO_2_ capture.

#### 2.3.3. Specific Surface Area and Micropore Surface Area

**Figure 11:**
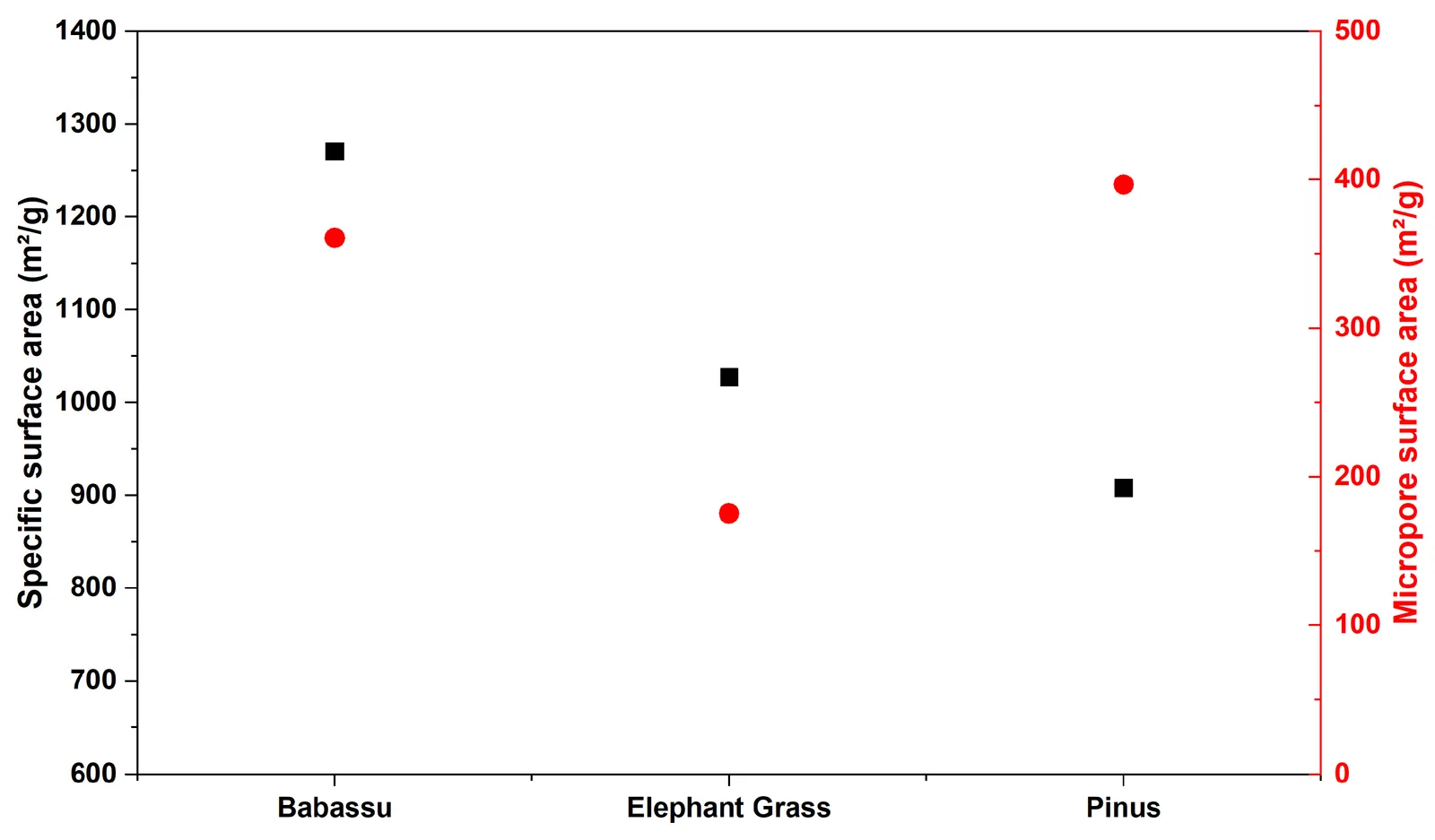
Specific surface area versus micropore area of steam-activated samples.

The specific surface area and micropore surface area results are presented in Figure 11. Babassu displayed the highest BET surface area, reaching 1270.53 m^2^ g^−1^, followed by Elephant Grass (1027.28 m^2^ g^−1^) and *Pinus* (907.87 m^2^ g^−1^). A similar trend was observed for micropore surface area, with values of about 360.83, 175.28, and 396.73 m^2^ g^−1^ for Babassu, Elephant Grass, and *Pinus*, respectively.

The material with the highest total surface area was not the one exhibiting the highest micropore surface area. Although Babassu showed the largest total surface area, *Pinus* exhibited the largest microporous surface.

This observation highlights an important aspect of porous carbon characterization: total surface area alone does not necessarily determine adsorption performance. A considerable fraction of the surface area generated during steam activation may originate from mesopores and wider pores, which contribute significantly to BET area but provide weaker adsorption potentials for CO_2_ molecules.

The relatively low micropore surface area observed for Elephant Grass further supports the hypothesis that steam activation promoted excessive pore widening in this material. The conversion of narrow micropores into larger pores decreases the contribution of microporous surfaces while increasing average pore diameter. By comparison, *Pinus* appears to retain a more favorable microporous architecture, despite possessing the lowest total BET surface area among the three materials.

#### 2.3.4. Maximum CO_2_ Adsorption Capacity and Adsorption Energy

**Figure 12:**
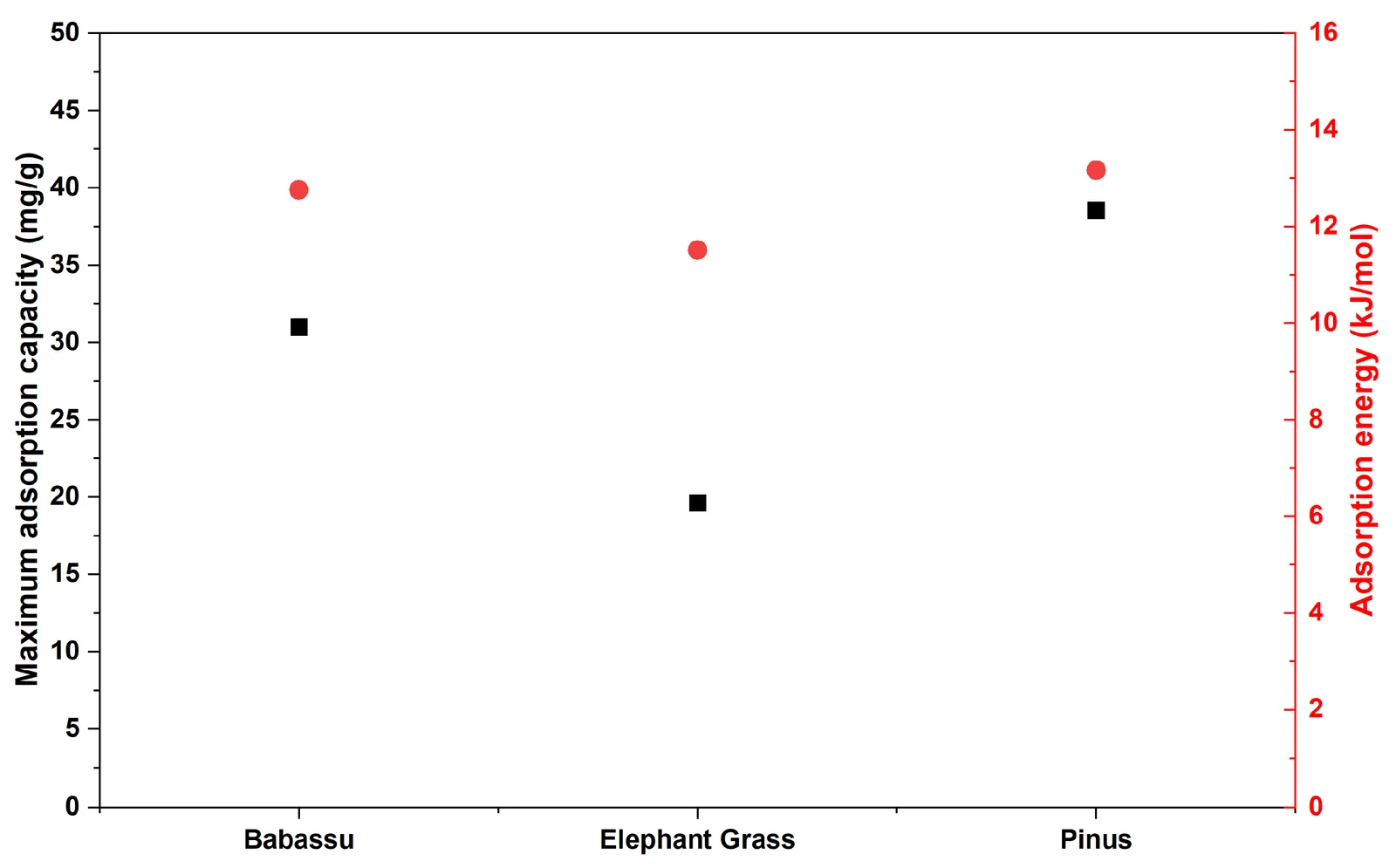
Maximum CO_2_ adsorption capacity versus adsorption energy of steam-activated samples.

Figure 12 presents the maximum adsorption capacity and adsorption energy obtained for the steam-activated biochars. *Pinus* exhibited the highest maximum adsorption capacity (38.54 mg g^−1^), followed by Babassu (31 mg g^−1^) and Elephant Grass (19.62 mg g^−1^). The adsorption energy followed the same tendency, reaching 13.163 kJ mol^−1^ for *Pinus*, 12.76 kJ mol^−1^ for Babassu, and 11.51 kJ mol^−1^ for Elephant Grass.

The similarity between the trends for adsorption energy and adsorption capacity suggests that the strength of CO_2_-surface interactions plays a major role in determining adsorption performance. The higher adsorption energy observed for *Pinus* indicates stronger confinement of CO_2_ molecules within narrow micropores, leading to greater adsorption effectiveness.

*Pinus* achieved the highest adsorption capacity despite exhibiting the lowest total BET surface area. This result clearly demonstrates that adsorption performance is governed more strongly by micropore characteristics than by overall surface area. The poor performance of Elephant Grass reinforces this interpretation. Although the material possessed a relatively high BET surface area exceeding 1000 m^2^ g^−1^, its low micropore volume and low micropore surface area resulted in significantly lower CO_2_ uptake. This finding indicates that much of the generated surface area was associated with larger pores that contribute little to adsorption under the investigated conditions.

Narrow micropores provide overlapping adsorption potentials that significantly enhance interactions between CO_2_ molecules and the carbon walls. Consequently, adsorbents with well-developed microporous networks generally display higher adsorption capacities than materials having predominantly mesoporous structures, even when the latter exhibit larger total surface areas.

The combined analysis of all parameters reveals a clear relationship between pore structure and adsorption performance. For *Pinus*, the combination of high micropore volume, high micropore surface area, narrow pore width, and the highest adsorption energy produced the best CO_2_ adsorption performance. Babassu exhibited intermediate behavior, balancing high total surface area with moderate microporosity. In contrast, Elephant Grass displayed evidence of excessive pore widening, resulting in lower micropore development and reduced adsorption efficiency.

Overall, the results indicate that steam activation successfully generated highly porous biochars; however, the effectiveness of the process for CO_2_ capture depended strongly on preserving narrow micropores. While larger pores contributed to high BET surface areas, the adsorption capacity was primarily controlled by the quantity and accessibility of microporous domains. Therefore, among the steam-activated materials, *Pinus* developed the most favorable pore architecture for CO_2_ adsorption, whereas Elephant Grass experienced greater pore widening and consequently lower adsorption performance.

A comparison of all textural parameters reveals that CO_2_ adsorption capacity was not directly controlled by BET surface area. For example, ACS B exhibited the highest BET surface area (1270.53 m^2^ g^−1^), but its adsorption capacity was considerably lower than that of the acid-washed KOH-activated samples. In contrast, ACKAW materials combined improved micropore accessibility, reduced inorganic blockage, and favorable adsorption energies, resulting in the highest adsorption capacities. These findings demonstrate that accessible microporosity and surface cleanliness are more important for CO_2_ capture than total surface area alone.

### 2.4. Adsorption–Desorption Cycles and Regenerability

**Figure 13:**
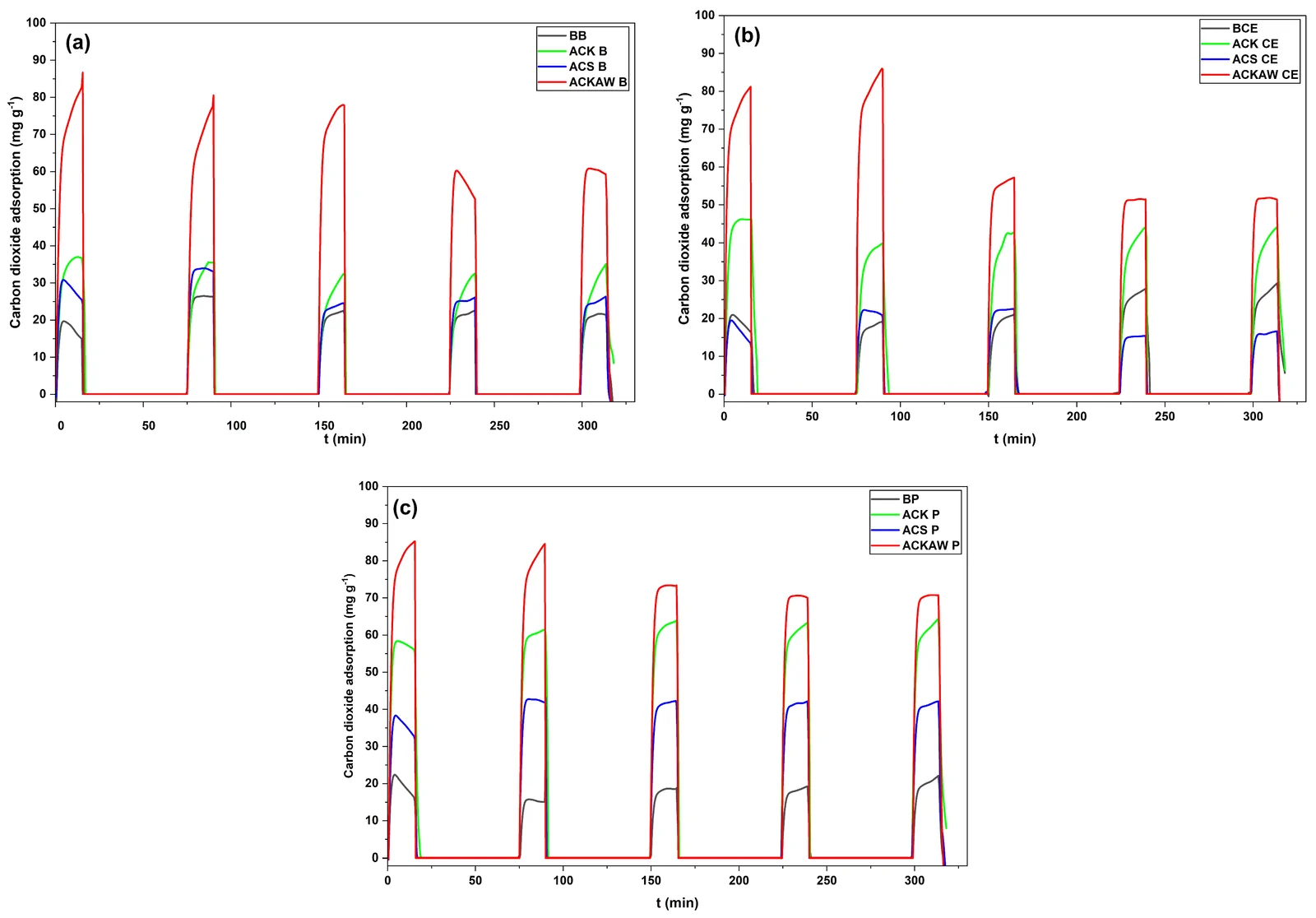
CO_2_ adsorption cycles for the babassu (**a**), elephant grass (**b**), and *Pinus* (**c**) samples.

The adsorption–desorption cycling experiments were performed to evaluate the stability and reusability of the biochars and activated carbons during repeated CO_2_ capture. Figure 13 presents the adsorption profiles obtained over five consecutive cycles for the materials derived from babassu, elephant grass, and *Pinus*. Overall, all samples exhibited reproducible adsorption–desorption behavior, indicating that the developed adsorbents possess adequate regeneration characteristics.

For all materials, the adsorption curves presented a rapid initial increase in CO_2_ uptake followed by a gradual approach to equilibrium. This behavior is characteristic of porous carbonaceous adsorbents and indicates that adsorption initially occurs at easily accessible sites located on the external surface and within larger pores. As these sites become occupied, mass transfer limitations within the microporous structure become more significant, reducing the adsorption rate until equilibrium is reached. During desorption, a rapid decrease in the adsorbed amount was observed, demonstrating efficient regeneration of the materials.

The biochars produced solely by pyrolysis exhibited the lowest adsorption capacities throughout the adsorption–desorption cycles. The limited performance of BB, BCE, and BP is associated with their low BET surface areas and the reduced development of micropores, which restrict the number of available adsorption sites.

Steam-activated materials generally exhibited improved cyclic performance compared with their corresponding biochars. The enhancement was particularly evident for *Pinus*-derived carbon (ACS P), which maintained adsorption capacities between approximately 38 and 43 mg g^−1^ throughout the cycles. This behavior reflects the highly developed porous network generated during steam activation and demonstrates the stability of the material under repeated adsorption and regeneration conditions.

The KOH-activated samples exhibited a further increase in adsorption performance, confirming the importance of micropore development for CO_2_ capture. The materials ACK B, ACK CE, and ACK P maintained adsorption capacities significantly higher than those of the original biochars, indicating that the pore structure generated during chemical activation remained stable throughout the cycling experiments.

The best cyclic performances were obtained for the samples subjected to KOH activation followed by HCl washing. ACKAW B, ACKAW CE, and ACKAW P exhibited the highest CO_2_ uptakes during the first adsorption cycle, reaching approximately 82.78, 81.19, and 85.24 mg g^−1^, respectively. Although a reduction in adsorption capacity was observed after the first cycle, all materials stabilized at relatively high adsorption levels during the subsequent cycles, demonstrating good regeneration behavior.

The decrease observed after the initial cycle was more pronounced for the elephant grass-derived material and may be related to the presence of highly energetic adsorption sites or ultramicroporous domains that undergo structural rearrangement during the first adsorption–desorption event. Nevertheless, the material maintained superior adsorption performance compared with the corresponding non-washed and steam-activated samples throughout all cycles.

Among all investigated materials, ACKAW P exhibited the highest stability, maintaining adsorption capacities between approximately 70 and 73 mg g^−1^ after the initial cycle. The lower performance loss suggests a more robust pore network and greater structural stability of the *Pinus*-derived carbon matrix.

## 3. Materials and Methods

### 3.1. Raw Materials

Three lignocellulosic biomasses were used as precursors for biochar production: babassu (*Attalea speciosa*) endocarp, elephant grass (*Pennisetum purpureum*), and *Pinus elliottii*. Elephant grass was cultivated at the Fazenda Souza experimental area of the University of Caxias do Sul (UCS, Brazil), while *Pinus* was obtained in pelletized form from commercial forestry residues. Babassu endocarp was supplied from the state of Maranhão, Brazil.

### 3.2. Biochar Production

Prior to pyrolysis, the biomasses were dried at 105 °C for 1 h to remove moisture. Biochars were produced by slow pyrolysis in a fixed-bed batch reactor under a nitrogen atmosphere. A mass of 30 g of biomass was used in each experiment. The reactor was heated from room temperature to 400 °C at a heating rate of 5 °C min^−1^ and maintained at the final temperature for 1 h. Nitrogen was continuously supplied at a flow rate of 100 mL min^−1^ to ensure an inert atmosphere throughout the process. The biochars obtained from babassu, elephant grass, and *Pinus* were designated as BB, BCE, and BP, respectively. The reactor used was described by [54].

### 3.3. Physical Activation

Physical activation was carried out using steam as activating agent. Initially, the biochars were ground and sieved, and the fraction passing through a 20-mesh sieve was used.

The samples were heated to 900 °C under nitrogen flow (30 mL min^−1^). After reaching the activation temperature, steam was injected at a rate between 1.3 and 1.5 g min^−1^ for 30 min. After activation, the reactor was cooled under nitrogen atmosphere. The activated samples were designated as ACS B, ACS CE, and ACS P.

### 3.4. Chemical Activation and Acid Washing

Chemical activation was performed using potassium hydroxide (KOH). Biochar and KOH were mixed at a mass ratio of 2:1 (biochar:KOH) in 100 mL of deionized water. The suspension was stirred at 200 rpm for 24 h and subsequently filtered and dried at 65 °C for 24 h.

The impregnated materials were then thermally treated at 800 °C for 2 h under a nitrogen atmosphere with a flow rate of 30 mL min^−1^ in a reactor described by [55]. After activation, the samples were washed in 100 mL of 2 mol L^−1^ hydrochloric acid (HCl) solution under agitation (200 rpm) for 48 h to remove residual inorganic compounds. The materials were then filtered, washed, and dried at 60 °C for 24 h.

The chemically activated samples were identified as ACK B, ACK CE, and ACK P, while the acid-washed materials were designated as ACKAW B, ACKAW CE, and ACKAW P.

### 3.5. Proximate Analysis

Moisture, volatile matter, ash content, and fixed carbon were determined according to ASTM D1762-84. All analyses were performed in triplicate. Fixed carbon was calculated by difference.

### 3.6. Textural Characterization

The specific surface area of the materials were determined by nitrogen adsorption–desorption isotherms using the Brunauer–Emmett–Teller (BET) method. Prior to analysis, the samples were degassed under nitrogen at 150 °C for 20 h. Measurements were performed using a NOVA 1200e surface area analyzer (Quantachrome Instruments, Boynton Beach, FL, USA).

Micropore parameters were calculated using the Dubinin–Radushkevich model, adsorption and desorption branches were obtained using carbon dioxide as the analysis gas. The fitting procedure provided the micropore volume, micropore surface area, average pore width, and characteristic adsorption energy for each sample.

### 3.7. Scanning Electron Microscopy and Energy-Dispersive Spectroscopy

Surface morphology was examined by scanning electron microscopy (SEM), and the semi-quantitative elemental composition was assessed by energy-dispersive X-ray spectroscopy (EDS). Prior to analysis, the samples were sputter-coated with a thin gold layer and analyzed using a MIRA 3 scanning electron microscope (TESCAN, Brno, Czech Republic).

### 3.8. CO_2_ Adsorption Measurements

CO_2_ adsorption experiments were performed using a thermogravimetric analyzer (STA 449 F3 Jupiter®, Netzsch, Selb, Germany). Approximately 10 mg of sample were heated to 105 °C under nitrogen atmosphere and maintained for 30 min to remove physically adsorbed moisture. Subsequently, the temperature was reduced to 25 °C, and the gas was switched from N_2_ to CO_2_ at a flow rate of 50 mL min^−1^. Adsorption was conducted for 15 min [42,49].

Adsorption–desorption cycling tests were carried out over five consecutive cycles. Desorption was performed by heating the sample from 25 to 105 °C at 10 °C min^−1^ under nitrogen atmosphere and maintaining this temperature for 5 min before beginning the next adsorption cycle.

## 4. Conclusions

The pyrolysis process produced biochar yields ranging from 25.68 to 33.04 wt.%, confirming the suitability of all three biomasses for the production of carbonaceous adsorbents. Among the produced biochars, *Pinus* and babassu exhibited the highest fixed-carbon contents and the lowest ash concentrations, indicating favorable characteristics for subsequent activation processes.

The activation treatments significantly modified the structure of the materials. Steam activation was the most effective route for the development of textural properties, producing specific surface areas of 1270.53, 1027.28, and 907.87 m^2^ g^−1^ for babassu, elephant grass, and *Pinus*, respectively. In contrast, chemical activation with KOH promoted substantial micropore development and pronounced morphological changes, particularly for the *Pinus*-derived material.

SEM revealed morphological changes, while EDS and ash-content analyses demonstrated that acid washing effectively removed potassium-containing compounds and other inorganic species remaining after chemical activation. Although the acid treatment reduced the measured BET surface area and total pore volume, it increased pore accessibility by removing mineral deposits that partially obstructed the porous network.

The CO_2_ adsorption results revealed that adsorption performance was not directly proportional to the BET surface area. Instead, micropore accessibility and surface cleanliness played a more important role in determining adsorption capacity. The highest adsorption performances were obtained for the samples activated with KOH and subsequently washed with HCl, reaching 82.78 mg g^−1^ for babassu, 81.19 mg g^−1^ for elephant grass, and 85.24 mg g^−1^ for *Pinus*. Adsorption–desorption cycling experiments confirmed the good regenerability of the developed materials, with adsorption capacities remaining stable over multiple cycles.

The results demonstrate that the combination of KOH activation and acid washing is an effective strategy for producing high-performance biochar-based adsorbents for CO_2_ capture. Among the evaluated materials, the *Pinus*-derived activated carbon exhibited the highest adsorption capacity, although all three biomass feedstock showed significant potential for the development of sustainable carbon capture materials.

## Figures and Tables

**Table 1:** Proximate analysis of biomass-derived biochars.

Sample	Moisture (%wt.)	Volatile Matter ^1^ (%wt.)	Ash ^1^ (%wt.)	Fixed Carbon ^2^ (%wt.)
BCE	8.09 ± 0.18	26.55 ± 1.91	18.43 ± 1.10	55.02 ± 1.97
BP	5.40 ± 0.20	27.18 ± 0.28	1.87 ± 0.01	70.99 ± 0.20
BB	6.09 ± 0.15	25.23 ± 2.31	4.71 ± 0.76	70.06 ± 3.08

^1^ dry basis. ^2^ by difference.

**Table 2:** Concentration of elements based on EDS.

Sample	Carbon (C)	Oxygen (O)	Silicon (Si)	Potassium (K)	Magnesium (Mg)	Calcium (Ca)
BB	79.9	15.9	2.6	1	0.1	n.d.
ACK B	59.2	22.4	0.6	9.6	1.4	3.4
ACKAW B	85.1	12.8	0.4	0.1	0.1	0.1
BCE	73.6	19.8	2.3	2.6	0.4	0.6
ACK CE	72.1	17.1	1.3	7.5	0.5	0.8
ACKAW CE	83.4	13.7	2	n.d.	0.1	0.1
BP	83.3	16.0	0.1	0.2	0.1	0.2
ACK P	75.3	15.2	0.1	8.4	0.1	0.5
ACKAW P	88.9	10.8	0.1	0.1	n.d	0.1

## Data Availability

Data available upon reasonable request.

## Supplementary Materials

The following supporting information can be downloaded at: https://www.mdpi.com/article/10.3390/molecules31172971/s1, Figure S1. EDS of samples BB (a), ACS B (b), ACK B (c) and ACKAW B (d); Figure S2. EDS of samples BCE (a), ACS CE (b), ACK CE (c) and ACK AW CE (d); Figure S3. EDS of samples BP (a), ACS P (b), ACK P (c) and ACKAW P (d); Figure S4. Micrographs of samples BCE (a), ACS CE (b), ACK CE (c) and ACKAW CE (d) at a magnitude of 10k×; Figure S5. Micrographs of samples BB (a), ACS B (b), ACK B (c) and ACKAW B (d) at a magnitude of 10k×; Figure S6. Micrographs of samples BP (a), ACS P (b), ACK P (c) and ACKAW P (d) at a magnitude of 10k×.

## Abbreviations

The following abbreviations are used in this manuscript:

BB	Biochar of babassu
ACK B	KOH-activated babassu biochar
ACKAW B	Babassu-derived activated carbon activated with KOH and washed with HCl
ACS B	Steam-activated babassu biochar
BCE	Biochar of elephant grass
ACK CE	KOH-activated elephant grass biochar
ACKAW CE	Elephant grass-derived activated carbon activated with KOH and washed with HCl
ACS CE	Steam-activated elephant grass biochar
BP	Biochar of *Pinus*
ACK P	KOH-activated *Pinus* biochar
ACKAW P	*Pinus*-derived activated carbon activated with KOH and washed with HCl
ACS P	Steam-activated *Pinus* biochar

## References

[1] Karimi M., Shirzad M., Silva J.A.C., Rodrigues A.E. (2022). Biomass/Biochar Carbon Materials for CO_2_ Capture and Sequestration by Cyclic Adsorption Processes: A Review and Prospects for Future Directions. J. CO2 Util..

[2] Liu C., Fu C., Li T., Zhang P., Xia Y., Wu Y., Lan Q., Li Y., Zhang Y., Gui J. (2023). CO_2_ Capture Using Biochar Derived from Conditioned Sludge via Pyrolysis. Sep. Purif. Technol..

[3] Oschatz M., Antonietti M. (2018). A Search for Selectivity to Enable CO_2_ Capture with Porous Adsorbents. Energy Environ. Sci..

[4] Shyam A., Ahmed K.R.A., Kumar J.P.N., Iniyan S., Goic R. (2025). Path of Carbon Dioxide Capture Technologies: An Overview. Next Sustain..

[5] Ji Y., Zhang C., Zhang X.J., Xie P.F., Wu C., Jiang L. (2022). A High Adsorption Capacity Bamboo Biochar for CO_2_ Capture for Low Temperature Heat Utilization. Sep. Purif. Technol..

[6] Cao L., Zhang X., Xu Y., Xiang W., Wang R., Ding F., Hong P., Gao B. (2022). Straw and Wood Based Biochar for CO_2_ Capture: Adsorption Performance and Governing Mechanisms. Sep. Purif. Technol..

[7] Deng H., Li T., Li H., Dang A., Han Y. (2024). Carbon-Based Adsorbents for CO_2_ Capture: A Systematic Review. J. Ind. Eng. Chem..

[8] Soo X.Y.D., Lee J.J.C., Wu W.Y., Tao L., Wang C., Zhu Q., Bu J. (2024). Advancements in CO_2_ Capture by Absorption and Adsorption: A Comprehensive Review. J. CO2 Util..

[9] Foorginezhad S., Weiland F., Chen Y., Hussain S., Ji X. (2025). Review and Analysis of Porous Adsorbents for Effective CO_2_ Capture. Renew. Sustain. Energy Rev..

[10] Wen C., Liu T., Wang D., Wang Y., Chen H., Luo G., Zhou Z., Li C., Xu M. (2023). Biochar as the Effective Adsorbent to Combustion Gaseous Pollutants: Preparation, Activation, Functionalization and the Adsorption Mechanisms. Prog. Energy Combust. Sci..

[11] Dissanayake P.D., You S., Igalavithana A.D., Xia Y., Bhatnagar A., Gupta S., Kua H.W., Kim S., Kwon J.H., Tsang D.C.W. (2020). Biochar-Based Adsorbents for Carbon Dioxide Capture: A Critical Review. Renew. Sustain. Energy Rev..

[12] Ferreira S.D., Manera C., Silvestre W.P., Pauletti G.F., Altafini C.R., Godinho M. (2019). Use of Biochar Produced from Elephant Grass by Pyrolysis in a Screw Reactor as a Soil Amendment. Waste Biomass Valorization.

[13] Jung S., Park Y.K., Kwon E.E. (2019). Strategic Use of Biochar for CO_2_ Capture and Sequestration. J. CO2 Util..

[14] Basu P. (2010). Biomass Gasification and Pyrolysis.

[15] Dhyani V., Bhaskar T. (2018). A Comprehensive Review on the Pyrolysis of Lignocellulosic Biomass. Renew. Energy.

[16] Nasriani H.R., Nasiri Ghiri M. (2026). Biomass Pyrolysis: Recent Advances in Characterisation and Energy Utilisation. Processes.

[17] Johannes L.P., Minh T.T.N., Xuan T.D. (2024). Elephant Grass (*Pennisetum purpureum*): A Bioenergy Resource Overview. Biomass.

[18] Ressiore C. A., Lima C.L.S., Turnhout E. (2024). Care Narratives: Babassu Breakers and Mother Palm Trees. Geoforum.

[19] Jang H.A., Pyo S.W., Choi Y.I., Lee H., Bae E.K., Ko J.H. (2026). Expanding the Research Frontiers of *Pinus* Species in Wood Biology. Forests.

[20] Bao Z., Shi C., Tu W., Li L., Li Q. (2022). Recent Developments in Modification of Biochar and Its Application in Soil Pollution Control and Ecoregulation. Environ. Pollut..

[21] Yao J., Wang Z., Liu M., Bai B., Zhang C. (2023). Nitrate-Nitrogen Adsorption Characteristics and Mechanisms of Various Garden Waste Biochars. Materials.

[22] Deng L., Xia W., Yang Z., Zhang W., Feng D., Sun S., Zhao Y. (2025). Research on Biochar Prepared by Trace KOH Catalyzed CO_2_ Activation vs KOH Activation as Advanced Candidate for Carbon Capture. Ranliao Huaxue Xuebao/J. Fuel Chem. Technol..

[23] Zhang L., Zuo S. (2024). The Significance of Lignocellulosic Raw Materials on the Pore Structure of Activated Carbons Prepared by Steam Activation. Molecules.

[24] Monteagudo J.M., Durán A., Alonso M., Stoica A.I. (2025). Investigation of Effectiveness of KOH-Activated Olive Pomace Biochar for Efficient Direct Air Capture of CO_2_. Sep. Purif. Technol..

[25] Zhang C., Sun S., He S., Wu C. (2022). Direct Air Capture of CO_2_ by KOH-Activated Bamboo Biochar. J. Energy Inst..

[26] Wang T., Quan X., Sun Z., Sun Z. (2025). Sustainable Biochar Synthesis via Synergistic H_2_O_2_-KOH Modification for Enhanced CO_2_ Physisorption. Carbon Capture Sci. Technol..

[27] Liu Y., Fang H., Zhang S., Wang C., Min L. (2025). Enhanced CO_2_ Adsorption of Alkali-Modified Sludge Biochar via Hydrothermal Activation: Mechanisms and Kinetics. Case Stud. Constr. Mater..

[28] Premchand P., Demichelis F., Galletti C., Chiaramonti D., Bensaid S., Antunes E., Fino D. (2024). Enhancing Biochar Production: A Technical Analysis of the Combined Influence of Chemical Activation (KOH and NaOH) and Pyrolysis Atmospheres (N_2_/CO_2_) on Yields and Properties of Rice Husk-Derived Biochar. J. Environ. Manag..

[29] Goel C., Mohan S., Dinesha P. (2021). CO_2_ Capture by Adsorption on Biomass-Derived Activated Char: A Review. Sci. Total Environ..

[30] Amer N.M., Lahijani P., Mohammadi M., Mohamed A.R. (2024). Modification of Biomass-Derived Biochar: A Practical Approach towards Development of Sustainable CO_2_ Adsorbent. Biomass Convers. Biorefin..

[31] Abbaspour N., Jordan C., Tondl G., Wąsik P., Gholizadeh T., Tomasetig D., Szlęk A., Pfeifer C., Harasek M., Korus A. (2025). Activated Biochars from Heavy Metal-Contaminated Biomass for CO_2_ Capture: Adsorption Performance and Dominant Mechanisms. J. CO2 Util..

[32] Hao W., Björkman E., Lilliestråle M., Hedin N. (2013). Activated Carbons Prepared from Hydrothermally Carbonized Waste Biomass Used as Adsorbents for CO_2_. Appl. Energy.

[33] Bonato J.L., Perondi D., Manera C., Borghetti M., Ferreira S.D., de Almeida Neuwald O., Godinho M. (2026). CO_2_ Adsorption Evaluation of Different Biochars Produced from Peanut Shell Pyrolysis. Biomass Convers. Biorefinery.

[34] Madzaki H., Karimghani W.A.W.A.B., Nurzalikharebitanim, Azilbaharialias (2016). Carbon Dioxide Adsorption on Sawdust Biochar. Procedia Engineering.

[35] Kwon C.W., Tae S., Mandal S. (2025). Comparative Analysis of CO_2_ Adsorption Performance of Bamboo and Orange Peel Biochars. Molecules.

[36] Oktaviana A.A., Hermana J., Syafei A.D., Hsi H.C. (2025). Effect of Pyrolysis Temperature of Domestic Sewage Sludge Biochar on CO_2_ Adsorption. Results Eng..

[37] Villardon A., Alcazar-Ruiz A., Dorado F., Sanchez-Silva L. (2024). Enhancing Carbon Dioxide Uptake in Biochar Derived from Husk Biomasses: Optimizing Biomass Particle Size and Steam Activation Conditions. J. Environ. Chem. Eng..

[38] Straka P., Cihlář J., Bičáková O. (2025). Production of Syngas and Hydrogen-Rich Gas from Lignocellulosic Biomass via Ru/Al_2_O_3_ Catalyst-Assisted Slow Pyrolysis. Catalysts.

[39] De Conto D., Silvestre W.P., Baldasso C., Godinho M. (2016). Performance of Rotary Kiln Reactor for the Elephant Grass Pyrolysis. Bioresour. Technol..

[40] Yang H., Yan R., Chen H., Lee D.H., Zheng C. (2007). Characteristics of Hemicellulose, Cellulose and Lignin Pyrolysis. Fuel.

[41] Ferreira S.D., Lazzarotto I.P., Junges J., Manera C., Godinho M., Osório E. (2017). Steam Gasification of Biochar Derived from Elephant Grass Pyrolysis in a Screw Reactor. Energy Convers. Manag..

[42] Botomé M.L., Poletto P., Junges J., Perondi D., Dettmer A., Godinho M. (2017). Preparation and Characterization of a Metal-Rich Activated Carbon from CCA-Treated Wood for CO_2_ Capture. Chem. Eng. J..

[43] Chang S., Wang L., Yao L. (2025). Properties of Sunflower Straw Biochar Activated Using Potassium Hydroxide. Molecules.

[44] Li K., Zhang D., Niu X., Guo H., Yu Y., Tang Z., Lin Z., Fu M. (2022). Insights into CO_2_ Adsorption on KOH-Activated Biochars Derived from the Mixed Sewage Sludge and Pine Sawdust. Sci. Total Environ..

[45] Zhang J., Zhang X., Li X., Song Z., Shao J., Zhang S., Yang H., Chen H. (2024). Prediction of CO_2_ Adsorption of Biochar under KOH Activation via Machine Learning. Carbon Capture Sci. Technol..

[46] Rabichi I., Ezzahi K., Yaacoubi F.E., Sekkouri C., Bouzid T., Ennaciri K., Ounas A., El Fels L., Hafidi M., Baçaoui A. (2025). Synthesis and Application of Biochar and KOH-Activated Carbon from Olive Mill Solid Waste for Polyphenol Removal. J. Mol. Liq..

[47] Zhao Y., Huang L., Sun G. (2025). Preparation of High-Performance KOH-Activated Biochar from Agricultural Waste (*Sapindus mukorossi*) and Its Application in Organic Dye Removal. Sustainability.

[48] Nazbakhsh M., Nabavi S.R., Jafarian S. (2025). Optimized Production of High-Performance Activated Biochar from Sugarcane Bagasse via Systematic Pyrolysis and Chemical Activation. Sustainability.

[49] Nunes I.d.S., Schnorr C., Perondi D., Godinho M., Diel J.C., Machado L.M.M., Dalla Nora F.B., Silva L.F.O., Dotto G.L. (2022). Valorization of Different Fractions from Butiá Pomace by Pyrolysis: H_2_ Generation and Use of the Biochars for CO_2_ Capture. Molecules.

[50] Zhang C., Sun S., Xu S., Wu C. (2022). CO_2_ Capture over Steam and KOH Activated Biochar: Effect of Relative Humidity. Biomass Bioenergy.

[51] Martín-Lara M.Á., Muñoz-Batista M.J., Blázquez G., Pereira L., Quintana A., Calero M. (2026). Turning Plastic and Biomass Waste into Adsorbents: CO_2_/CH_4_ Separation and Comparison with Commercial Carbons. Waste Manag..

[52] Zhang C., Ji Y., Li C., Zhang Y., Sun S., Xu Y., Jiang L., Wu C. (2023). The Application of Biochar for CO_2_ Capture: Influence of Biochar Preparation and CO_2_ Capture Reactors. Ind. Eng. Chem. Res..

[53] Dantas S., Struckhoff K.C., Thommes M., Neimark A.V. (2021). Pore Size Characterization of Micro-Mesoporous Carbons Using CO_2_ Adsorption. Carbon.

[54] Manera C., Perondi D., Barcellos T., Godinho M. (2018). CO_2_ Gasification of Elephant Grass in a Fixed Bed Reactor. Sci. Cum Ind..

[55] Biondo L.D., Manera C., Aguzzoli C., Godinho M. (2024). DoE-Driven Thermodynamic Assessment of COX-Free Hydrogen Production from Methane Decomposition. Catal. Commun..

