# Supplementary material for: Biomass-Derived Activated Biochars to CO2 Adsorption
Source: Molecules. 2026 Aug 25;31(17):2971. doi: 10.3390/molecules31172971 (PMC13567300; doi:10.3390/molecules31172971)
Supplement: Supplementary file 1 [file molecules-31-02971-s001.zip › molecules-4502978-supplementary.pdf]

Supplementary Morphological and Elemental Characterization

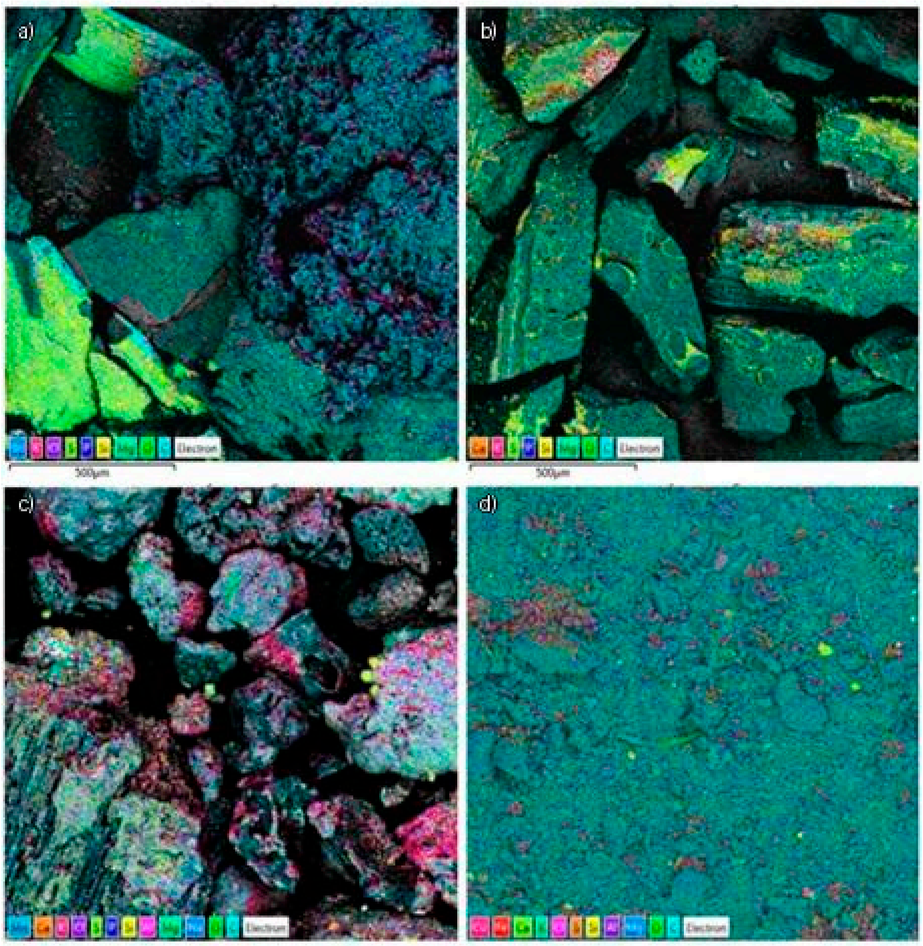

Figure S1 - EDS of samples BB a), ACS B b), ACK B c) and ACKAW B d)

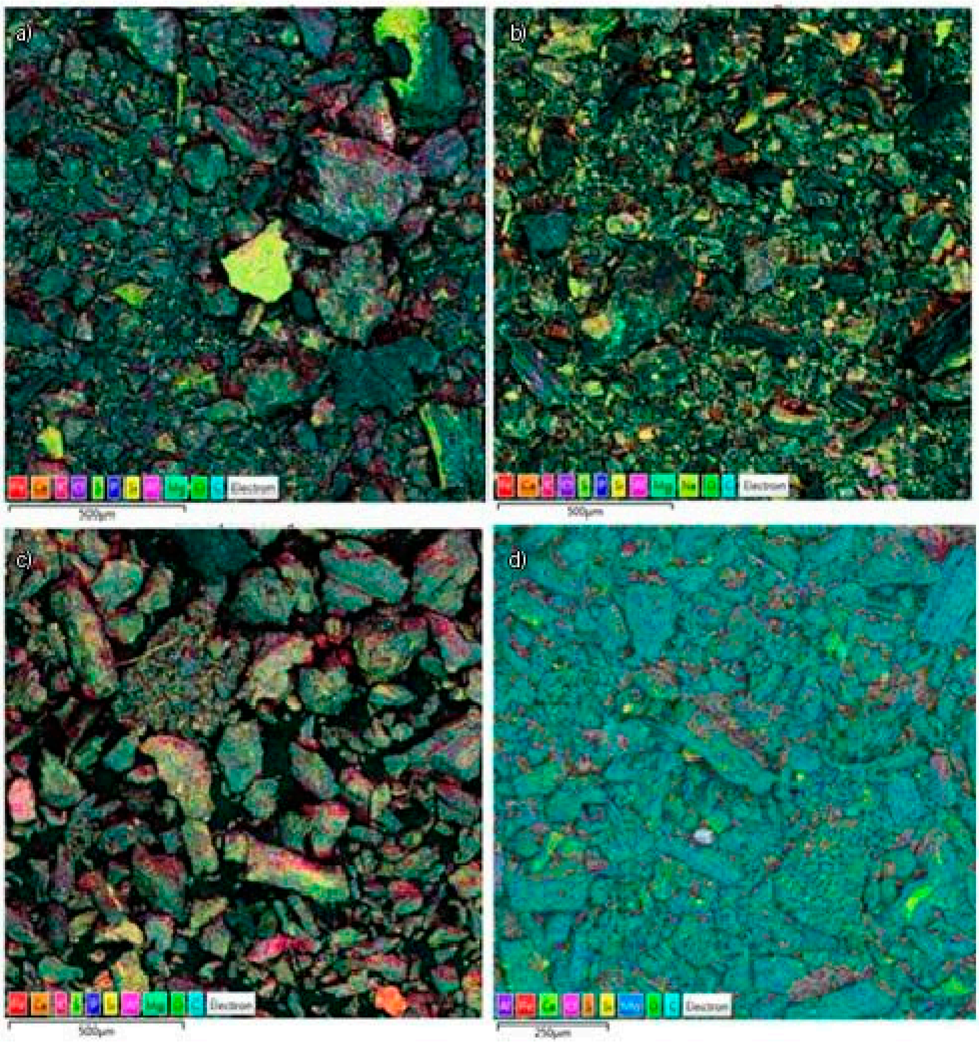

Figure S2 - EDS of samples BCE a), ACS CE b), ACK CE c) and ACK AW CE d).

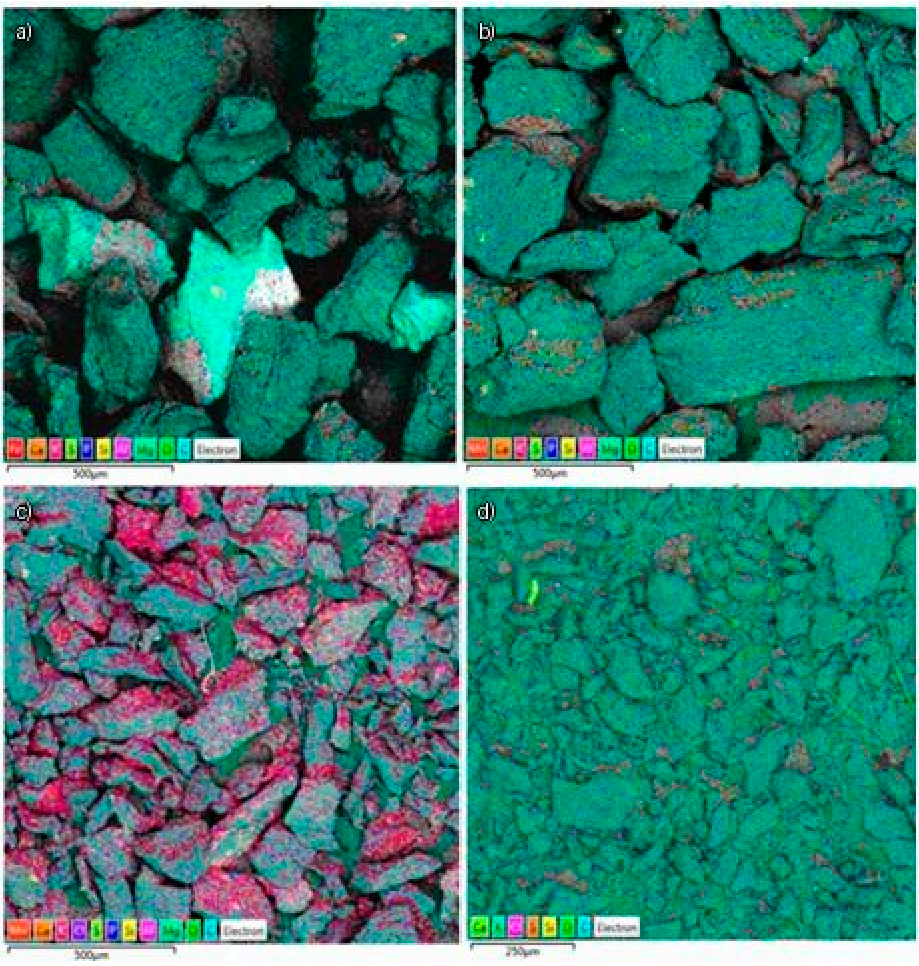

Figure S3 - EDS of samples BP a), ACS P b), ACK P c) and ACKAW P d)

S1.1. Morphological and Elemental Characterization

Scanning electron microscopy (SEM) and energy-dispersive spectroscopy (EDS) were employed to investigate the structural and compositional changes induced by steam activation, KOH activation, and subsequent acid washing. The morphological evolution observed for the three biomass-derived materials provides important insights into the mechanisms responsible for the development of porosity and the enhancement of CO_2 adsorption performance.

S1.1.1. Elephant Grass Materials

SEM micrographs of the elephant grass biochar and activated carbons are presented in Figure S4. The original biochar (BCE) exhibited a morphology typical of lignocellulosic materials subjected to pyrolysis, preserving part of the native plant structure. Fibrous domains, longitudinal channels, and interconnected macropores were observed, together with a relatively smooth surface. These characteristics indicate that pyrolysis at 400 °C promoted carbonization while maintaining much of the original biomass architecture.

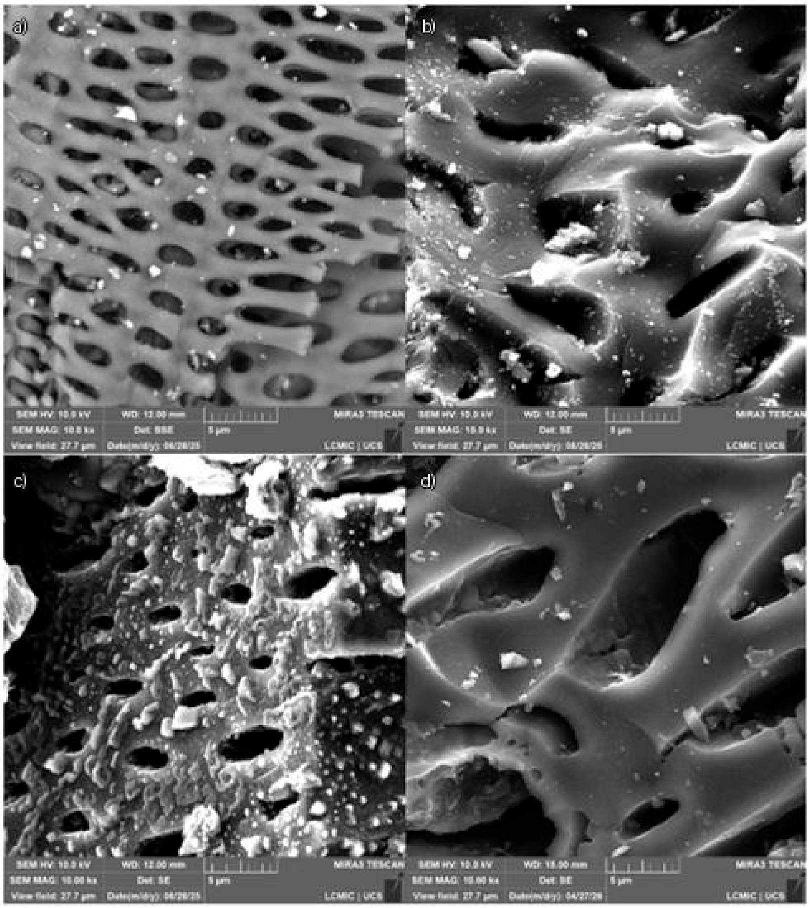

Figure S4 - Micrographs of samples BCE a), ACS CE b), ACK CE c) and ACKAW CE d) at a magnitude of 10kx.

After steam activation (ACS CE), an enlargement of pre-existing pores and an increase in surface roughness were observed. The appearance of deeper cavities suggests that water vapor gasification selectively removed carbon from the solid matrix, contributing to the development of additional porosity while preserving a significant portion of the fibrous structure.

More pronounced modifications were observed after chemical activation with KOH (ACK CE). The surface became considerably rougher, with the appearance of numerous cavities and localized fragmentation of the carbon matrix. These changes are consistent with the reaction of KOH with carbon promotes structural modification and pore development. The development of microporosity is confirmed by the CO_2 adsorption (Dubinin–Radushkevich) analysis presented in the manuscript. Compared with steam activation, the chemical activation route produced substantially greater morphological modifications.

The acid-washed sample (ACKAW CE) exhibited a cleaner and more homogeneous surface, with pores becoming more visible and better defined. The removal of inorganic residues generated during chemical activation appears to have exposed previously obstructed pores, increased pore accessibility and potentially contributing to the adsorption behavior discussed later.

S1.1.2. Babassu Materials

The SEM images of the babassu-derived materials are presented in Figure S5. Unlike elephant grass biochar, BB exhibited a more heterogeneous and partially collapsed structure, characterized by irregular cavities and a less organized porous network. These features reflect the intrinsic structure of babassu endocarp and the transformations occurring during pyrolysis.

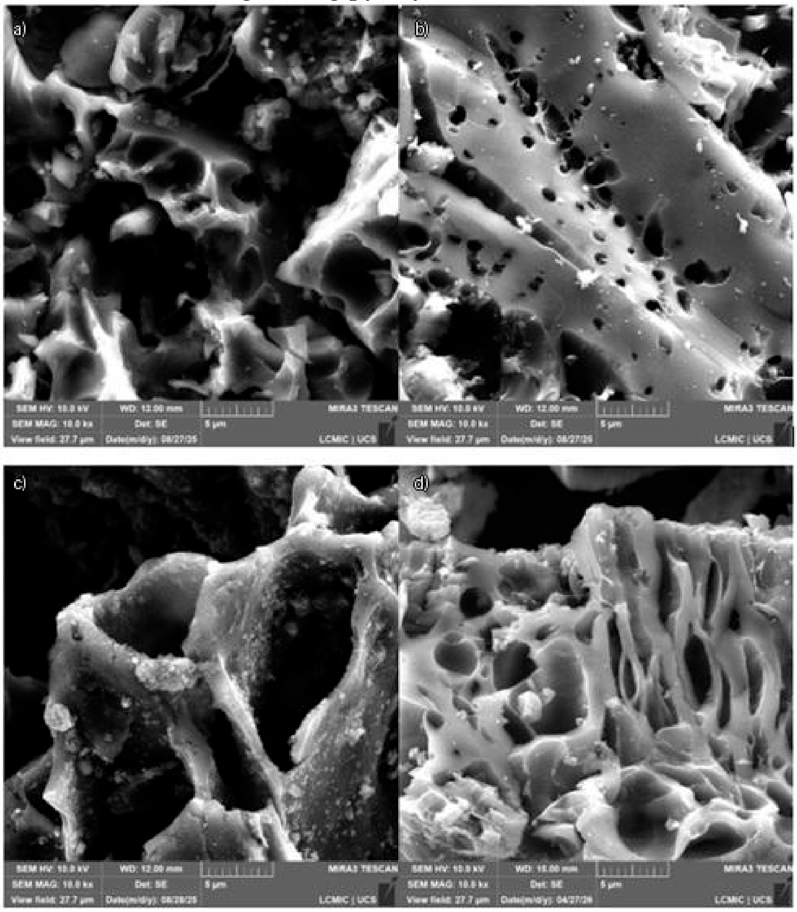

Figure S5 - Micrographs of samples BB a), ACS B b), ACK B c) and ACKAW B d) at a magnitude of 10kx.

Steam activation (ACS B) promoted the development of pores distributed non-uniformly throughout the surface and increased surface roughness. The observed changes indicate that gasification occurred heterogeneously within the carbon matrix, creating localized porous regions without drastically altering the overall morphology of the material.

KOH activation resulted in more intense structural modifications. The sample ACK B presented extensive fragmentation of the carbon matrix, the formation of numerous microcavities, and regions with partial structural collapse. These features are characteristic of aggressive chemical activation and indicate significant carbon removal and pore generation. Although structural disruption was observed, such modifications are generally associated with the creation of a larger accessible pore network.

Following acid washing, the surface of ACKAW B became noticeably cleaner, with more clearly defined pores and fewer surface deposits. The removal of inorganic compounds generated during KOH activation likely contributed to the opening of previously blocked pores, exposing a larger fraction of the internal porous structure.

S1.1.3. Pinus Materials

Figure S6 shows the SEM micrographs of the pinus-derived materials. The untreated biochar (BP) exhibited a relatively compact and smooth surface with a limited number of visible pores. This morphology suggests that pyrolysis at 400 °C mainly promoted carbonization while generating only a modest degree of pore development.

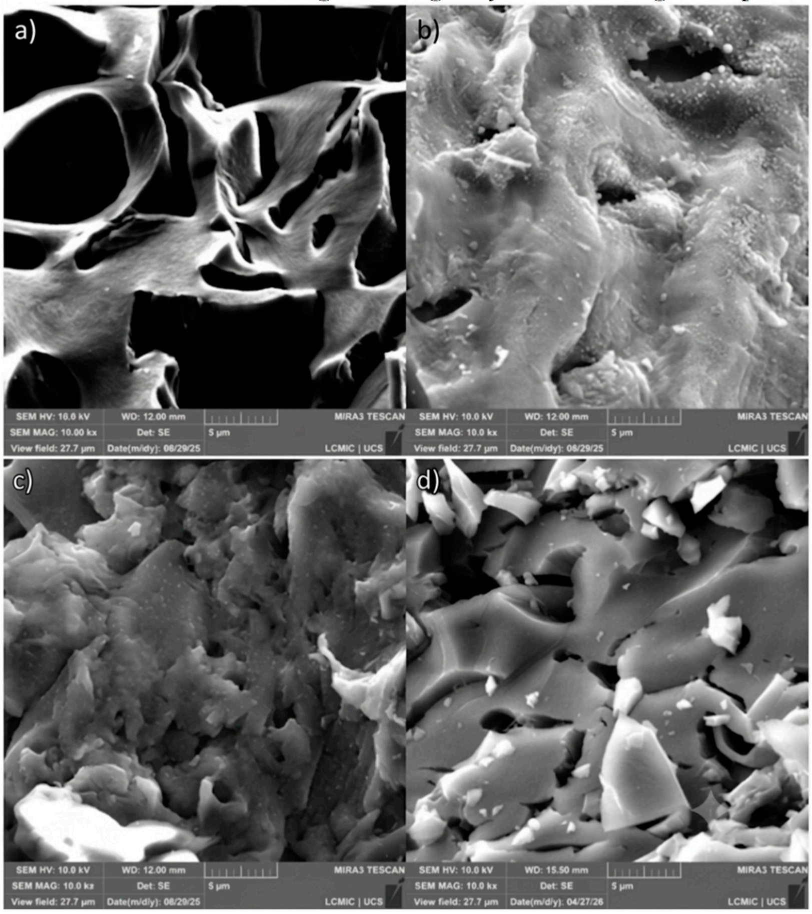

Figure S6 - Micrographs of samples BP a), ACS P b), ACK P c) and ACKAW P d) at a magnitude of 10kx.

Steam activation (ACS P) produced moderate changes in surface morphology. Small cavities appeared throughout the surface and a slight increase in roughness was observed, indicating the onset of pore development through carbon gasification. However, the overall integrity of the carbon matrix remained largely preserved

Chemical activation with KOH induced substantially greater changes. The activated material exhibited a highly irregular surface, extensive pore formation, and a marked increase in structural heterogeneity. These observations suggesting that KOH activation greatly enhanced the development of the porous structure. The formation of microporosity was confirmed by the textural characterization presented in the manuscript.

After acid washing, the resulting material (ACKAW P) displayed a cleaner surface and more evident pore openings. The elimination of residual potassium compounds and other inorganic species contributed to the exposure of the internal porous structure and improved accessibility to adsorption sites. These characteristics are consistent with the superior adsorption performance obtained for this material.
